# Biofortified indica rice attains iron and zinc nutrition dietary targets in the field

**DOI:** 10.1038/srep19792

**Published:** 2016-01-25

**Authors:** Kurniawan R. Trijatmiko, Conrado Dueñas, Nikolaos Tsakirpaloglou, Lina Torrizo, Felichi Mae Arines, Cheryl Adeva, Jeanette Balindong, Norman Oliva, Maria V. Sapasap, Jaime Borrero, Jessica Rey, Perigio Francisco, Andy Nelson, Hiromi Nakanishi, Enzo Lombi, Elad Tako, Raymond P. Glahn, James Stangoulis, Prabhjit Chadha-Mohanty, Alexander A. T. Johnson, Joe Tohme, Gerard Barry, Inez H. Slamet-Loedin

**Affiliations:** 1 Plant Breeding, Genetics, and Biotechnology Division, International Rice Research Institute, DAPO Box 7777, Metro Manila, Philippines; 2 Centro Internacional de Agricultura Tropical, Cali, Colombia; 3 Social Sciences Division, International Rice Research Institute, DAPO Box 7777, Metro Manila, Philippines; 4 Faculty of Geo-Information and Earth Observation (ITC), University of Twente, Enschede 7500 AE, The Netherlands; 5 Department of Global Agricultural Sciences, Graduate School of Agricultural and Life Sciences, The University of Tokyo, 1-1-1 Yayoi, Bunkyo-ku, Tokyo 113-8657 Japan; 6 Centre for Environmental Risk Assessment and Remediation, University of South Australia, Australia; 7 United States Department of Agriculture-Agricultural Research Service, Robert W. Holley Center for Agriculture and Health, Cornell University, New York; 8 School of Biological Sciences, Flinders University of South Australia, Adelaide, Australia; 9 School of Botany, The University of Melbourne, Victoria 3010, Australia; 10 Indonesian Center for Agricultural Biotechnology and Genetic Resources Research and Development, Bogor 16111, Indonesia; 11 Research Center for Biotechnology, Indonesian Institute of Sciences, Cibinong 16911, Indonesia

## Abstract

More than two billion people are micronutrient deficient. Polished grains of popular rice varieties have concentration of approximately 2 μg g^−1^ iron (Fe) and 16 μg g^−1 ^zinc (Zn). The HarvestPlus breeding programs for biofortified rice target 13 μg g^−1 ^Fe and 28 μg g^−1 ^Zn to reach approximately 30% of the estimated average requirement (EAR). Reports on engineering Fe content in rice have shown an increase up to 18 μg g^−1^ in glasshouse settings; in contrast, under field conditions, 4 μg g^−1^ was the highest reported concentration. Here, we report on selected transgenic events, field evaluated in two countries, showing 15 μg g^−1 ^Fe and 45.7 μg g^−1 ^Zn in polished grain. Rigorous selection was applied to 1,689 IR64 transgenic events for insert cleanliness and, trait and agronomic performances. Event NASFer-274 containing rice nicotianamine synthase (*OsNAS2*) and soybean ferritin (*SferH-1*) genes showed a single locus insertion without a yield penalty or altered grain quality. Endosperm Fe and Zn enrichment was visualized by X-ray fluorescence imaging. The Caco-2 cell assay indicated that Fe is bioavailable. No harmful heavy metals were detected in the grain. The trait remained stable in different genotype backgrounds.

Micronutrient deficiencies or “hidden hunger” affect about 38% of pregnant women and 43% of preschool children worldwide and are most prevalent in developing countries^1^. More than 30% of the world’s population is anemic^2^. Global studies estimate that approximately half of this is due to iron-deficiency anemia (IDA)^3^. IDA can affect productivity and cause serious health consequences, including impaired cognitive development in children, a weakened immune system, and increased risk of morbidity^4^.

Zinc deficiency is a major cause of stunting among children^5^. About 165 million children with stunted growth run a risk of compromised cognitive development and physical capability^6^,^7^. Biofortification, the delivery of micronutrients via staple food crops, has been proposed to complement existing efforts for the alleviation of micronutrient deficiency^8^.

Conventional breeding efforts for developing Fe-enriched polished rice face major challenges because of the limited variability in Fe concentration in polished grains among rice germplasm^9^. Engineering Fe- and Zn-biofortified rice has been extensively reviewed^10^,^11^. However, 15 years after the pioneering attempt^12^ and numerous conventional breeding efforts^9^, reaching the 30% EAR nutritional targets for iron and zinc concentrations in polished rice grains^8^ still remains a major challenge^10^. This 30% EAR was calculated as 13 μg g^−1 ^Fe and 28 μg g^−1^ Zn in polished grains taking into account of 90% micronutrient retention after processing and 10% bioavailability for Fe and 25% bioavailability for Zn^8^. These levels have been recommended by nutritionists to achieve potential biological impact in adult women and children.

The highest grain Fe concentration reported in glasshouse studies of genetically modified (GM) temperate japonica rice was a 4.2-fold increase^13^, reaching 18 μg g^−1^ from a baseline of 4.5 μg g^−1^, while a maximum of a 6-fold increase was achieved^14^ from a baseline of 1.2 μg g^−1^ to 7 μg g^−1^. An extensive study on the overexpression of three iron homeostasis genes^15^ successfully obtained 8 μg g^−1^ in the glasshouse; however, in confined field trials (CFT), the concentration declined to 4 μg g^−1^, emphasizing the need for further improvement. Field trials are indispensable as recently reported^16^ to provide more realistic achievements closer to actual farming conditions. Maintaining the high-yield trait of the background popular genotype is crucial for farmer adoption. In addition, maintaining high yield could avoid selection of improved high-micronutrient lines due to concentration effect. As shown in wheat, grain Zn and grain yield concentration are often inversely correlated^17^. The high micronutrient grain concentration could be a bias when plant yield and milling degree are lower than in the comparative popular varieties.

Application of basic science achievements to the agricultural sector, particularly in the field of genetic engineering, is often underestimated. A large number of events need to be produced in order to apply rigorous selection^18^ to obtain transgenic events suitable for deregulation and farmer adoption. Our aim is to develop low-cost inbred biofortified (Fe-/Zn-dense) rice in popular indica varieties by translating the advances of fundamental science^12^,^13^,^14^,^15^ into a potential product. The product concept consists of achieving the Fe and Zn dietary target 14  μg g^−1^ from the baseline of 2  μg g^−1^ in the field, well-defined single-locus transgene integration, a good and stable agronomic performance under field settings. Limited number of thorough translational science studies has hindered GM crop development in the public sector.

We pursued the sink and source strategy by co-expressing *Sfer-H1* and *OsNAS2* genes in mega-variety IR64 and evaluated several combinations of promoters and transgenes. Ferritin is an iron storage protein capable of binding up to 4,500 Fe atoms per molecule^19^, and a major source of iron in vegetarian diets^20^. Ferritin in legumes has a long history of safe consumption; thus, it represents an attractive target protein for improving rice grain Fe concentration. Based on our earlier report^21^, we selected *SferH-1* gene instead of using *SferH-2* gene which encodes a more stable protein^22^.

In rice, iron is absorbed either directly or as a complex chelated by mugineic acid phytosiderophores^23^ such as 2′-deoxymugeneic acid (DMA). Biosynthesis of nicotianamine (NA), a precursor of DMA, is catalyzed by NA synthase^24^ (NAS). Different NAS genes have been used to develop GM rice^13^,^14^,^15^. Three NAS genes (*OsNAS1*, *OsNAS2*, and *OsNAS3*) have been identified in rice^24^. We selected *OsNAS2* among the orthologues because its overexpression has been shown to be more effective for rice grain Fe enhancement^13^. For the selectable marker, we used *aph4* gene-encoding enzyme hygromycin B phosphotransferase^25^. Foods containing this protein have been approved for markets in nine countries including USA, Canada, Japan, Australia, South Korea, New Zealand, Taiwan, Mexico and Indonesia^26^.

Here, we report on the development and characterization of potential candidate Fe- and Zn-dense transgenic events with a novel combination of promoter and gene orthologues for future release. This is the first report on achieving Fe and Zn biofortification nutrition targets in rice under field conditions. For broader impact, we introduced the genes to the widely grown IR64 indica cultivar, and bred the trait into other popular rice cultivars from South and Southeast Asia where Fe and Zn deficiencies are prevalent. Fe- and Zn-dense rice could eventually contribute to improve life quality in rural areas and a reduction in significant economic losses due to micronutrient deficiency^27^.

## Results

### Generation and selection of events

We generated global maps from updated data on micronutrient deficiency, rice consumption, and poverty distribution^2^,^7^,^28^,^29^,^30^ to highlight the strong interconnection of micronutrient deficiency, poverty rate, and rice consumption (see Supplementary Fig. S1 online). An unequivocal overlap between all three of these issues is observed across the maps.

**Figure 1:**
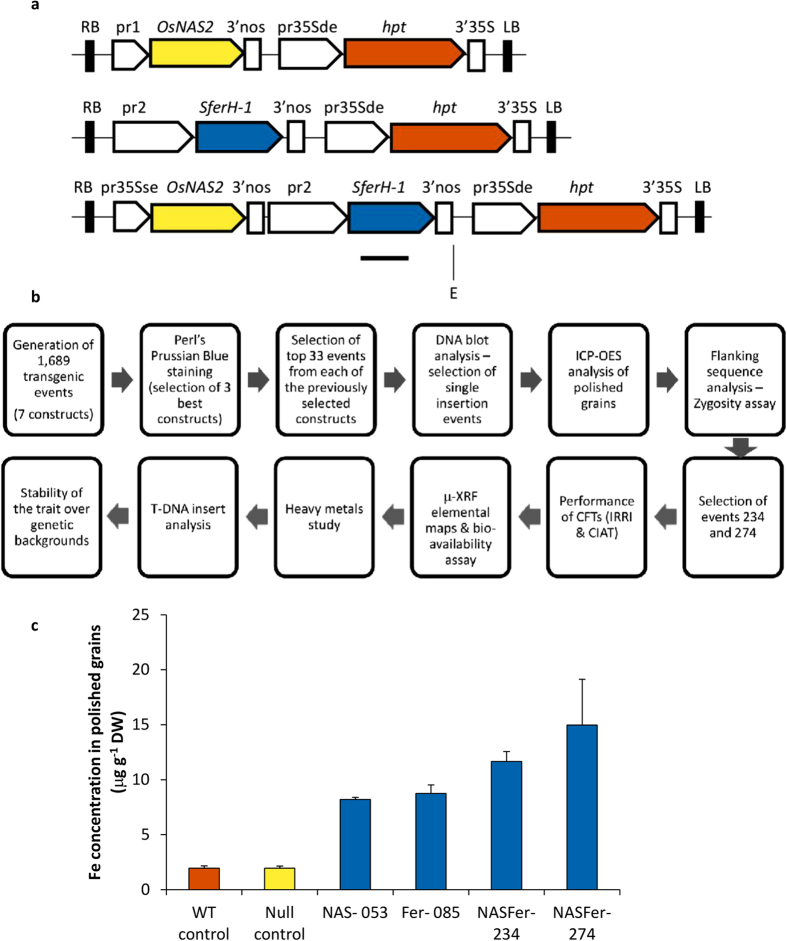
Strategy for the development of biofortified high-iron rice and the Fe concentration achieved in T_2_ polished seeds. (**a**) Schematic diagram of the T-DNA constructs prepared for rice transformation for the purposes of our study. RB and LB represent the right and left borders of the T-DNA, respectively; pr1: represents the constitutive promoters pr35S with single enhancer (pr35Sse), maize ubiquitin promoter, or rice endosperm-specific promoter glutelinB1; pr2: represents the rice endosperm-specific promoters glutelinB1 or glutelinA2; pr35Sde represents the constitutive promoter pr35S with double enhancer; *OsNAS2*: *Oryza sativa* nicotianamine synthase 2; *SferH-1*: *Glycine max* ferritin subunit H-1; *hpt*: hygromycin phosphotransferase; 3′nos: 3′UTR of nopaline synthase; 3′35S: 3′UTR of 35S cauliflower mosaic virus gene. The horizontal line below *SferH-1* represents the deduced hybridization position of the digoxigenin-labeled cDNA probe; E: *Eco*RI. (**b**) Flow-chart displaying the steps that have been followed for the development of biofortified high-Fe/-Zn rice. (**c**) Fe concentration (μg g^−1^ DW) of polished seeds harvested from T_1_ homozygous plants of representative NAS, Fer, and NASFer events, null segregant, and non-transformed rice under screenhouse conditions. Bars represent the means ± s.d. of three replicates.

To select a product with the desirable trait of Fe- and Zn-dense rice and robust field performance, we generated and screened 1,689 independent IR64 transgenic events obtained through transformation of seven constructs containing Fe storage and/or chelator genes driven by various promoters (Fig. 1a, Supplementary Table S1 online). The flow of the entire screening process and validation strategy is presented in Fig. 1b.

We prioritized our selection based on the polished grain iron concentration. The highest number of plants showing intense staining when Perls’ Prussian blue was used in T_1_ grain sections was obtained from three constructs containing *OsNAS*2 under control of a 35S promoter and/or soybean ferritin under control of a glutelinA2 promoter^31^ (Supplementary Table S1 online). The transformed plants derived from the construct containing *GluA2::SferH-1* in combination with *35S:OsNAS2* (Supplementary Table S1 online, coded as IRS495 or NASFer) gave the highest number of plants with intense staining, higher than the similar construct in which *SferH-1* is driven by glutelinB1 promoter (IRS493, Supplementary Table S1 online).

We selected up to 33 events from each construct with the most intense Fe staining (Perls’ Prussian blue) for copy number analysis (Supplementary Table S2 online). A majority of the transgenic events with intense staining contained multiple inserts, but events/lines with single-locus insertion were also identified (Supplementary Table S2 online). To accelerate the selection process, homozygous lines were selected in the segregating T_1_ generation using a multiplexed PCR assay with three oligonucleotide primers (Supplementary Fig. S2 online) on selected events with high Fe concentration and have a single insert of the three selected constructs (Supplementary Table S2 online). The elemental analysis on homozygous T_2_ polished seeds using inductively coupled plasma-optical emission spectrometry (ICP-OES) showed a significant 7.5-fold increase in Fe concentration, reaching 15 μg g^−1^ from the 2 μg g^−1^ baseline in the non-transformed IR64 control (Fig. 1c). This level of Fe concentration in polished grains was achieved in plants generated using the NASFer construct. Using the single-gene approach of *OsNAS2* or *SferH-1*, the maximum Fe concentration achieved in this study was 8.8 μg g^−1^ (Supplementary Table S2 online).

**Figure 2:**
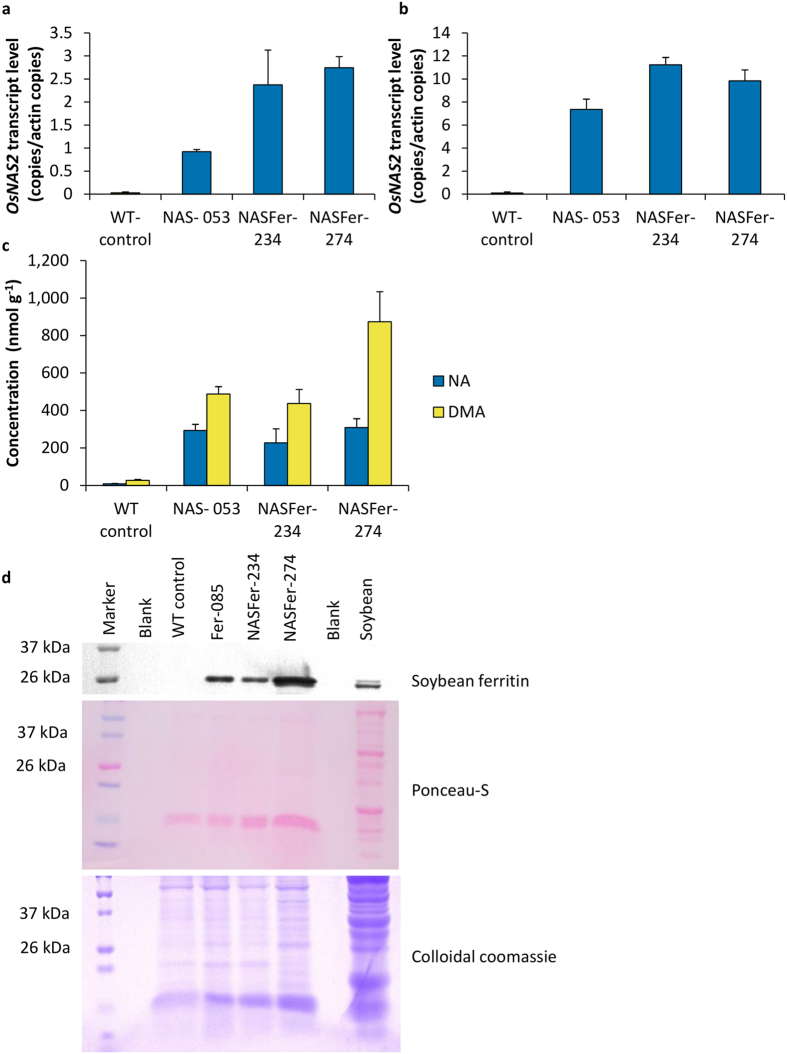
Expression of transgenes in the representative events. (**a**) Relative quantification of *OsNAS2* transcript levels of representative NAS and NASFer events and non-transformed rice in the roots of 8 days old seedlings. Bars represent the means ± s.d. of three biological replicates, each with three technical replicates of real-time RT-PCR. (**b**) Relative quantification of *OsNAS2* transcript levels of representative NAS and NASFer events and non-transformed rice in the leaves of 8 days old seedlings. Bars represent the means ± s.d. of three biological replicates, each with three technical replicates of real-time RT-PCR. (**c**) NA and DMA concentrations in T_2_ homozygous polished seeds of representative NAS and NASFer events and non-transformed rice. Bars represent the means ± s.d. of three replicates. (**d**) Immunoblot analysis of soybean ferritin protein in T_2_ polished seeds of representative Fer and NASFer events, non-transformed rice, and soybean. Ponceau-S staining of the membrane and colloidal coomassie staining of the gel served as loading controls.

Quantitative RT-PCR confirmed enhanced expression of *OsNAS2* in roots and leaves of transgenic events (Fig. 2a,b). We also observed enhanced expression of *OsNAAT1* (Nicotianamine Aminotransferase 1) and *OsDMAS1* (Deoxymugineic Acid Synthase 1) genes in the transgenic events (Supplementary Fig. S3 online). The enhanced expressions of these three genes involved in deoxymugineic acid (DMA) biosynthesis lead to significant increase of NA and DMA concentrations in polished grains of transgenic events by up to thirty two- and thirty three-fold, respectively, compared to WT (Fig. 2c). Accumulation of ferritin in polished grains was detected by immunoblot assay (Fig. 2d).

### Field Trials in the Philippines and Colombia

**Figure 3:**
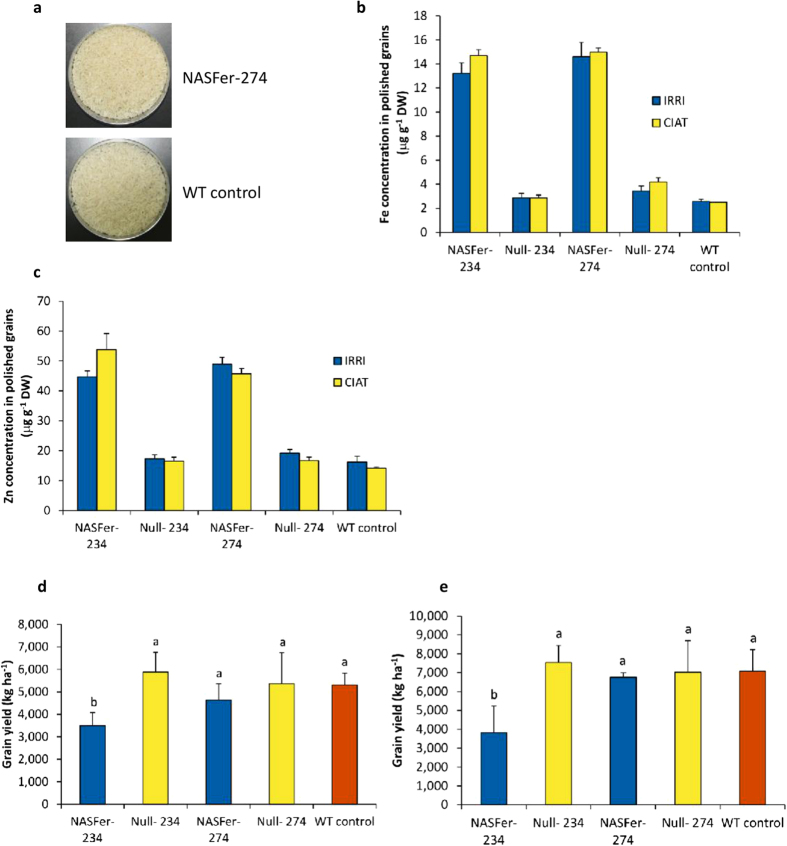
Field trials for evaluation of target trait and agronomic characters of two lead events. (**a**) Polished grains of lead event NASFer-274 and non-transformed rice. (**b**) Fe concentration (μg g^−1 ^DW) of T_3_ polished seeds of lead events NASFer-234 and NASFer-274 under field conditions. Bars represent the means ± s.d. of three replicates. (**c**) Zn concentration (μg g^−1 ^DW) of T_3_ polished seeds of lead events NASFer-234 and NASFer-274 under field conditions. Bars represent the means ± s.d. of three replicates. (**d**) Grain yield (kg ha^−1^) of T_3_ seeds of lead events NASFer-234 and NASFer-274 under field condition at IRRI. Bars represent the means ± s.d. of four replicates. Means labeled with different letters (**a,b**) differ significantly at the 5% level. (**e**) Grain yield (kg ha^−1^) of T_3_ seeds of lead events NASFer-234 and NASFer-274 under field condition at CIAT. Bars represent the means ± s.d. of four replicates. Means labeled with different letters (**a,b**) differ significantly at the 5% level.

Two high-Fe events with a single insert (NASFer-234 and NASFer-274) and no backbone integration beyond the T-DNA borders (Supplementary Table S2 online) were evaluated in CFTs at IRRI-Philippines and CIAT-Colombia (Supplementary Fig. S4 online). Figure 3a shows that the rice samples were well milled after 2.5 minutes of milling by a Kett Mill laboratory milling machine. The absence of bran in our polished rice was confirmed by microscopy observation (Supplementary Fig. S5 online). We achieved the Fe target of 14.6–15.0 and 13.2–14.7 μg g^−1^ (a 6-fold increase) for NASFer-274 and NASFer-234, respectively, in both locations (Fig. 3b). Additionally, these two transgenic events accumulated 2.7- to 3.8-fold more Zn when compared with the wild-type IR64 in both locations (Fig. 3c). This simultaneous increase in both Fe and Zn in single grains could have tremendous potential for alleviating both deficiencies.

Agronomic evaluation of the event NASFer-274 showed no yield penalty at both IRRI-Philippines and CIAT-Colombia (Fig. 3d,e), whereas the event NASFer-234 exhibited a lower yield in both locations when compared with IR64 and the null (negative segregant or azygous) (Fig. 3d,e). Grain quality testing on the most promising event (NASFer-274) did not reveal any difference in protein content, amylose content, gel consistency, seed size, and chalkiness (Supplementary Table S3 online).

### Elemental maps revealed significant increases of Fe and Zn localization in NASFer-274 endosperm

**Figure 4:**
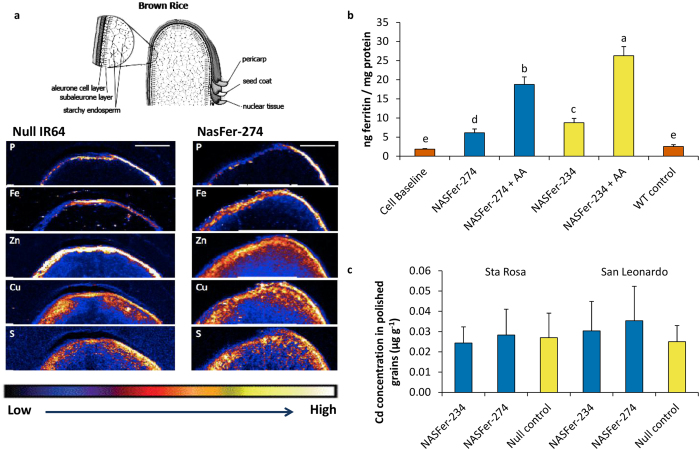
Characterization of lead events. (**a**) Grain elemental maps of brown grains of homozygous transgenic and null of NASFer-274. The color scale represents different elemental accumulations with black and white corresponding to the lowest and highest accumulations, respectively. The circled area in the diagram of brown rice (whole grain) indicates the grain layers presented in the XFM maps. (**b**) Fe bioavailability in grains of transgenic events NASFer-234 and NASFer-274 and a wild type (WT) in the absence/presence of ascorbic acid. Bars represent the means ± s.d. of three replicates. Means labeled with different letters (**a–e**) differ significantly at the 5% level. (**c**) Cd concentrations of polished seeds of transgenic events NASFer-234 and NASFer-274, nulls, and a wild type (WT) grown in high-Cd soils. Bars represent the means ± s.d. of three replicates.

Synchrotron X-ray fluorescence microscopy (XFM) was used to map the localization of several metal cations transported by NA (Fe, Zn, Cu) as well as elements indicative of phytic acid and protein (P and S, respectively) in transversal sections of null and lead event NASFer-274 whole grain (Fig. 4a). The elemental maps corresponded well with the ICP-OES elemental results (Supplementary Table S4 online) and demonstrated that the grain of event NASFer-274 had higher localization of Fe in aleurone, sub-aleurone and outer endosperm layers relative to null grain. The Zn maps indicated less accumulation in the aleurone and much higher Zn localization in the sub-aleurone and outer endosperm layers of NASFer-274 grain. The localization pattern of P did not differ between NASFer-274 and null grain, indicating no difference in phytate distribution between the two grain types. The S elemental maps, by contrast, showed slightly higher endosperm localization in NASFer-274 grain and, in conjunction with the ICP-OES elemental results, suggest that protein may be slightly increased in NASFer-274 endosperm. The Cu elemental maps also indicated slightly higher Cu localization in NASFer-274 endosperm.

### Enhanced Fe in the events is bioavailable

*In vitro* measurement of bioavailability in the new Fe-enhanced crops is important to predict level of Fe absorption in human. *In vitro* digestion/Caco-2 cell culture assays using T_4_ polished grains showed an increase in Fe availability for both of our lead events; this increased bioavailability was more pronounced in the presence of ascorbic acid (Fig. 4b and Supplementary Table S5 online). Ascorbic acid was reported as the most efficient enhancer of the absorption of non-heme Fe^32^.

### Increased NA concentrations in the grain did not enhance grain heavy metal accumulation

Our data showed that cadmium (Cd), arsenic (As), and lead (Pb) concentrations in polished grains harvested from two CFTs were below detection limits by ICP-OES (Supplementary Table S4 online), indicating that increased NA concentrations in the grain did not enhance grain heavy metal accumulation. To further test the extent of possible Cd accumulation, high-Fe/-Zn rice lines were planted in pots containing Cd-contaminated soil from two different locations (0.104 and 0.246 μg g^−1^ available Cd, respectively). A more sensitive ICP-MS (mass-spectrometry) analysis of harvested polished grains showed no significant difference between transgenic plants and controls, and all transgenic plants had Cd concentrations of <0.05 μg g^−1^ (Fig. 4c).

### Characterization of integration sites of two lead events

**Figure 5:**
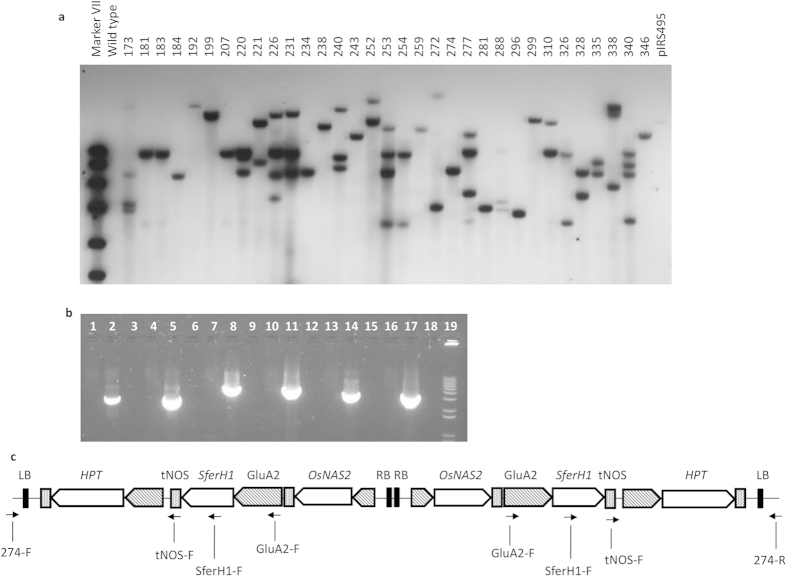
Characterization of integration site of lead event. (**a**) Genomic DNA blot hybridization from T_0_ and non-transformed rice plants to determine the T-DNA insertion number of selected NASFer events. (**b**) PCR using non-transformed rice and lead event NASFer-274 with primer pairs tNOS-F and 274-upstream-flanking (lanes 1 and 2, expected product size: 2,877 bp), tNOS-F and 274-downstream-flanking (lanes 4 and 5, expected product size: 2,835 bp), GluA2-F and 274-upstream-flanking (lanes 7 and 8, expected product size: 4,664 bp), GluA2-F and 274-downstream-flanking (lanes 10 and 11, expected product size: 4,622 bp), SferH-1-RT-F and 274-upstream-flanking (lanes 13 and 14, expected product size: 3,517 bp), SferH-1-RT-F and 274-downstream-flanking (lanes 16 and 17, expected product size: 3,475 bp), blank (lanes 3, 6, 9, 12, 15, 18), and molecular weight markers (lane 19, 1 kb plus DNA ladder). (**c**) Schematic diagram of inverted T-DNA repeat configuration of lead event NASFer-274.

DNA blot analysis using a single-cutter endonuclease within the T-DNA indicated single-copy insertions in our two lead events (Fig. 5a). Nevertheless, PCR-based assay analysis and sequencing indicated integration of two T-DNA copies oriented as an inverted repeat in both events (Fig. 5b,c, Supplementary Fig. S2 online, Supplementary Fig. S6 online, Supplementary Fig. S7 online). In agreement with previous studies^33^, the inverted T-DNA repeat of the two selected events did not trigger transgene silencing as shown by transcript expression and immunoblot analyses (Fig. 2a,b,d).

### Trait stability in different genotype backgrounds

**Figure 6:**
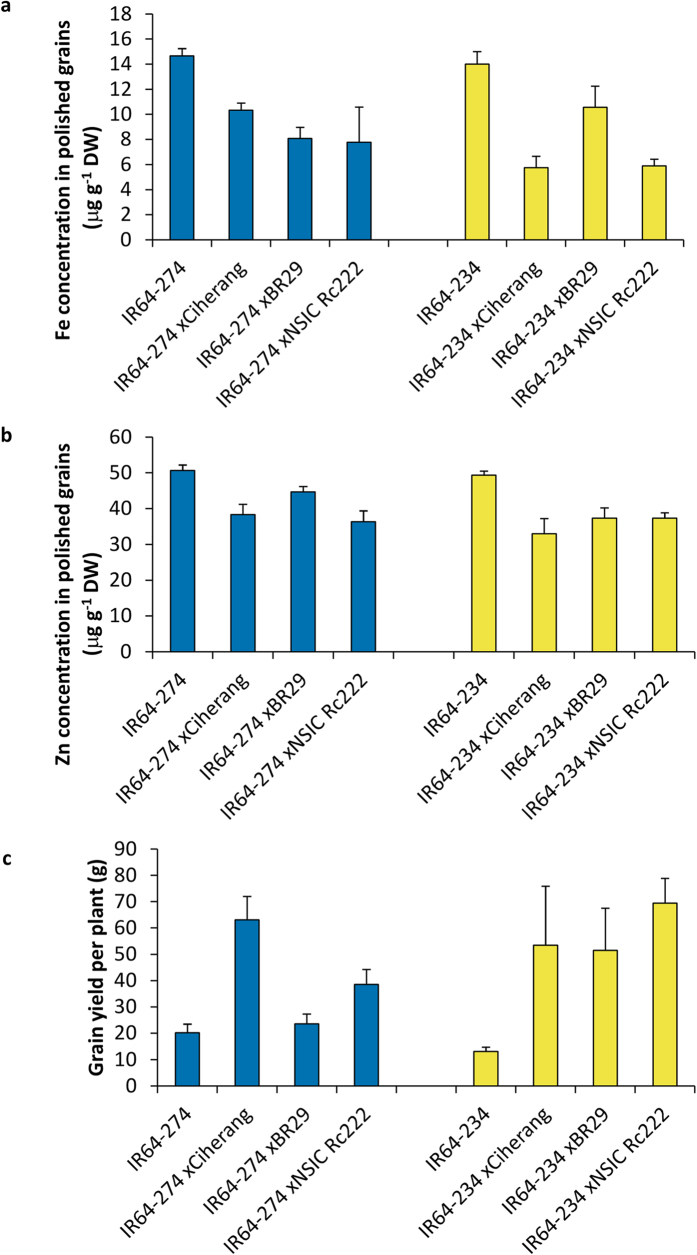
Stability of Fe and Zn concentrations in different genetic backgrounds. (**a**) Fe concentration (μg g^−1 ^DW) of polished seeds harvested from F_1_ plants derived from crossing between lead events and popular varieties. (**b**) Zn concentration (μg g^−1 ^DW) of polished seeds harvested from F_1_ plants derived from crossing between lead events and popular varieties. (**c**) Grain yield of F_1_ plants derived from crossing between lead event and popular varieties. Bars represent the means ± s.d. of three replicates.

The two selected events (274 and 234) were crossed with farmers’ popular varieties from the Philippines (NSic Rc222), Indonesia (Ciherang), and Bangladesh (BR29). The ICP-OES Fe elemental measurement, carried out in the F_2_ segregant grains, to gain an indication of trait stability in different genotypes showed concentrations ranging from 6 to 11 μg g^−1^ for Fe (Fig. 6a) and from 33 to 45 μg g^−1^ for Zn (Fig. 6b). This slight reduction, from 15 μg g^−1^ in homozygous material to 11 μg g^−1^, is expected since the F_2_ bulk polished grains were segregated grains derived from heterozygous parents. In addition, the yield per plant of the crosses with event NASFer-274 increased compared to the IR64 control, and was even higher in the event NASFer-234 (Fig. 6c) in this F_1_ population; therefore, the grain mineral concentration was most likely diluted because of the higher yield. Actual yield data should be obtained under field conditions on advanced backcross generations. We evaluated the Fe-polished seeds to obtain an indication of the trait stability. These data confirm the trend that this trait in general remains stable in different genotype backgrounds, even though there is some variation.

## Discussion

More than three billion people consume rice as staple food, with an average consumption of 75.20 kg/capita/year^28^,^29^. Moreover, in countries with medium to high prevalence of Fe and Zn deficiencies^2^,^7^, polished rice consumption jumps up to 150 kg/capita/year. This shows the vast potential of biofortified rice to serve as a micronutrient-enriched product to alleviate the severe micronutrient deficiency in rural and urban populations with limited purchasing power and limited access to more diverse diets.

Our comprehensive studies focused on translating the state-of-the-art research on iron and zinc homeostasis genes^10^,^11^ needed to generate biofortified rice for potential release. We first over expressed NAS single transgene in indica rice, but the maximum Fe concentration in polished grain was only 8.2 μg g^−1^ (Fig. 1c, Supplementary Table S2 online), lower than the concentration obtained previously in japonica rice^13^. This could be due to the difference in the varietal background, the favorable glasshouse versus screenhouse conditions, or plant yield dilution factor. Additionally, the baseline of grain Fe concentrations in the reported study^13^ was 4.5 μg g^−1^, higher than the 2–3 μg g^−1^ general baseline of popular indica varieties^8^. Recent evaluation across 1,763 rice germplasm accessions showed that grain Fe concentration is generally higher in the japonica subgroup^34^. We used indica rice since it is the popular varietal group in the target countries^35^.

Under field conditions, only two single-insert events from 1,689 generated events, reached the biofortification target to potentially fulfill the minimum of 30% of estimated average human requirement for Fe and Zn (Fig. 3b,c). This emphasizes the utmost importance of multiple construct evaluations and production of a large number of transgenic events to allow robust selection.

The two events with highest micronutrient concentrations in this study have different combination of promoters and gene orthologues than previously reported^10^,^11^. Here we selected the *SferH-1* gene, instead of using *SferH-2* gene which encodes a more stable protein^22^. Degradation of ferritin and the release of iron during cooking and gastric digestion are important for human absorption^36^ and food safety. The *SferH-1* of the best performed events were driven by the GlutelinA2 (GluA2) promoter. GluA2 promoter directs strong expression as early as 7 DAF^37^ (Supplementary Fig. S8 online), whereas Globulin 1 (Glb1) promoter directs strong expression mainly at 17 DAF^38^. Translocation of Fe from leaves is highly dependent on the concurrent phloem loading of the other major assimilates such as sucrose^39^. Since the highest rate of sucrose unloading into developing grain occurs at 6–12 days after flowering^40^, likewise for Fe, accumulation of ferritin protein at early grain-filling would be beneficial to capture the Fe unloaded during this period. Furthermore, a recent study showed that, at 10 DAF, the central endosperm enters a programmed cell death stage and, by 16 DAF, the cells in the central region die^41^.

It is also important to note that our initial screening was performed in the screenhouse with paddy soil to minimize the glasshouse microclimate effects on the grain Fe and Zn. The genetic and environmental conditions play major roles for both Zn and Fe concentration in the grain. It was reported that concentration achieved in the glasshouse decreased when evaluated under field conditions^15^. In addition, grain Fe concentration was higher in unflooded conditions than in flooded conditions^34^. Field experiments are important because phenotyping under favorable conditions in tightly controlled pot experiment often do not represent conditions prevailing in the field, although it is useful for potential candidate gene assessment^42^,^43^.

Event NASFer-234 showed a yield penalty in the field trials, whereas no yield penalty was observed in NASFer-274 (Fig. 3d,e). One possible reason is endogenous gene disruption (knockout) since analysis of insertion sites showed that, in NASFer-234, T-DNA was integrated in the second exon of an endogenous gene on chromosome 6 (Supplementary Fig. S6 online). On the other hand, in NASFer-274, T-DNA was inserted in the 3′UTR of a gene on chromosome 3 (Supplementary Fig. S7 online), therefore it is not likely to disturb the function of the endogenous gene. Insertion after the stop codon in *Arabidopsis* rarely affects the expression of a disrupted genes^44^, which probably causes the absence of a yield penalty in event 274.

Further investigation of the insertion site showed that both events have two copies in one locus (Fig. 5b,c, Supplementary Fig. S2 online, Supplementary Fig. S6 online, Supplementary Fig. S7 online), indicating a gene dosage effect aside from position effect. This emphasizes the need for multiple transgene insertions to elevate iron up to the target concentration, which is in line with other efforts using multiple transgenes to reach a 6-fold Fe increase^14^,^15^. Having two copies of the same transgenes, encoding for two distinct proteins, instead of four different proteins represents an advantage, since the protein safety dossier studies required for biosafety deregulation would be fewer.

Since Zn-associated transporters can also co-transport Zn-mimic Cd^45^ we measured the concentration of Cd in the polished grains (Fig. 4c). Using ICP-OES, all transgenic plants grown in IRRI’s CFTs had negligible Cd or other heavy metals. Concomitantly, in high-cadmium soil, there was no significant difference between NASFer-274 and -234 and their IR64 null (azygous) control counterparts, and the concentration is far below the Codex Alimentarius^46^ threshold concentration of 0.4 μg g^−1^. Similar to our findings, high-Zn japonica rice overexpressing *NAS* genes did not show an increase in grain Cd^11^,^47^, suggesting that NA is highly specific in chelating zinc over cadmium^45^. However, further studies need to be performed to compare cultivation in flooded and upland conditions in order to give farming system recommendations to farmers in the future.

To effectively improve human nutritional status, it is essential for Fe and Zn in biofortified staple food to be bioavailable. Enhancement in Fe concentration in the biofortified crop may not be translated proportionally in similar increase of absorbed Fe, because of the possible simultaneous increase of Fe inhibitors or enhancers in the new product^48^. The *in vitro* screening employs a simulated gastric and intestinal digestion of food coupled with culture of human intestinal cells to indicate Fe biovailability in human intestine^48^,^49^. Here our result on Caco-2 cell assay revealed increased Fe bioavailability in both lead transgenic events (Fig. 4b), suggesting limited increase on Fe absorption inhibitors.

The values of grain Fe and Zn concentrations of the crossing material with other popular cultivars obtained in our study (Fig. 6a,b) indicated that the trait is potentially stable in multiple backgrounds. This is crucial for future breeding applications in different countries.

### Concluding remarks

We have successfully accomplished the proof of concept on attaining Fe/Zn nutritional targets under flooded field conditions to fulfill 30% of EAR in the human diet in a well-characterized GM event of the widely consumed indica rice cultivar without a yield penalty. This achievement enables the future option to combine Fe, Zn and provitamin A traits in rice grain. The high-Fe and Zn rice could complement other current micronutrient intervention strategies such as supplementation and food fortification to alleviate nutritional deficiencies in rural regions and in urban poverty-stricken populations where these interventions are less effective.

## Methods

### Generation of plant transformation vectors and transgenic rice

Construction of plant transformation vectors is described in the Supplementary Methods. Transformation of indica rice cv. IR64 was performed using an immature embryo as previously described^50^. Transgenic plants were grown in greenhouse conditions. The presence of a transgene in the transformants was confirmed by PCR as described in the Supplementary Methods.

### Selection of best constructs and events based on Fe staining

A semi-quantitative analysis of Fe concentration^51^ with brief modifications was performed on brown seeds for the mass screening of the generated transgenic events to select the best construct as described in the Supplementary Methods. Subsequently, selection of best events from the best constructs were performed using Perl’s Prussian blue staining on polished seeds as described in the Supplementary Methods.

### Measurement of iron concentration in T_1_ and T_2_ grains

Polished grain samples (0.600–0.625 g) were digested in an ultrapure HNO_3_–HClO_4_ mixture in a microwave autoclave (Ultra Clave II, MLS GmbH, Leutkirch, Germany). Iron and zinc concentrations were determined using ICP-OES (Perkin Elmer ICP Optima 5300DV). The National Institute of Standards and Technology (NIST) rice flour standard 1568a and the Wageningen Evaluating Programs for Analytical Laboratories (WEPAL) IPE-135 were used as quality controls, and the samples were digested and analyzed using the same method as used for the rice samples.

### DNA blot analysis

Total DNA was prepared from leaves of T_0_ plants as previously described^53^. EcoRI-digested DNA (10 μg) was separated by agarose gel electrophoresis, blotted to a nylon membrane, and hybridized with a SferH-1 (for IRS491 and IRS495) or hpt (for IRS433) probe labeled with digoxigenin (Roche Applied Science, Germany). Hybridization and detection were carried out as previously described^54^.

### T-DNA flanking sequence recovery

Genomic sequences flanking the T-DNA left border were amplified using inverse PCR or TAIL-PCR as described in the Supplementary Methods. Transgenic event-specific amplification products were excised from the agarose, purified using GeneClean Kit II (QBiogene), cloned into pGEM-T Easy (Promega), and sequenced. Identification of the insert position in the rice genome was performed using a BlastN algorithm^55^ at the National Center for Biotechnology Information (www.ncbi.nlm.nih.gov).

### Zygosity test of T_1_ plants

Genomic DNA was isolated from leaves of T_1_ plants as previously described^53^. A PCR-based assay using three oligonucleotides was performed as previously described^18^ to determine the zygosity of the T_1_ plants.

### Amplification and sequencing for confirmation of T-DNA inverted repeat configuration in event NASFer-274

A primer that is complementary to a genetic element within T-DNA insert was paired with either primer that is complementary to the upstream flanking sequence or primer that is complementary to the downstream flanking sequence to amplify genomic DNA of a homozygous T_1_ NASFer-274 line with non-transformed IR64 as a negative control as described in the Supplementary Methods. Amplification products were excised from the agarose, purified using GeneClean Kit II (QBiogene), cloned into pGEM-T Easy (Promega), and sequenced.

### Relative quantification of transcript

Total RNA was isolated from leaves of wild-type and transgenic rice plants using Plant RNA Purification Reagent (Invitrogen, Carlsbad, California, USA) and purified using RNeasy Mini Kit (Qiagen, Valencia, California, USA). Real-time quantitative RT-PCR (qRT-PCR) was performed as described in the Supplementary Methods. The transcript level of the OsNAS2 transgene and endogenous OsNAAT1 and OsDMAS1 in transgenic and WT were normalized to the expression level of actin, using the relative standard curve method as previously described^15^.

### Immunoblot analysis

Grain protein was isolated as previously described^21^. Protein concentration was determined by the BCA method (Pierce, Rockford, IL). Protein samples were separated by SDS-PAGE, transferred to Invitrolon PVDF Filter Paper Sandwich membrane (Life Technologies, CA), and immunoblotted with a polyclonal anti-soybean ferritin^56^ at 1:2000 dilution. The anti-rabbit IgG-horseradish peroxidase (Bio-Rad) at 1:1000 dilution was used according to the manufacturer’s instructions for secondary detection of the antibodies. The activity of horseradish peroxidase linked to the secondary antibody was detected with 4-chloro-1-naphthol colorimetric substrate (Bio-Rad).

### Determination of the NA concentration

T_2_ seeds harvested from homozygous transgenic T_1_ plants were analyzed for NA and DMA concentrations using the LC/ESI-TOF-MS method as previously described^57^.

### Synchrotron X-ray fluorescence microscopy (XFM)

The grain elemental maps of the homozygous transgenic and null of NASFer-274 were collected at the XFM beamline at the Australian Synchrotron as previously described^13^.

### Field evaluation

Confined field trials were conducted at IRRI-Philippines and CIAT-Colombia. The trials were planted in a randomized complete block design with four replications. Plot size was 4 rows x 15 hills (25 cm between rows and 20 cm between hills) with one plant per hill. Chemical properties of the soil at each CFT are presented in Supplementary Table S6 and S7 online. For IRRI CFT, NPKZn fertilizers were applied at a rate of 150-30-30-5 kg ha^−1^. While P, K and Zn were applied in full before leveling, N was applied in three splits, once before leveling and twice as top dressing. For CIAT CFT, NPKZnFe fertilizers were applied at a rate of 400-130-220-26-104 kg ha^−1^. While P, Zn and Fe were applied in full at the time of transplanting, N was applied in three splits, and K was applied in two splits. Six plants from each plot were randomly chosen for the evaluation of grain yield. The seeds of the six plants were then bulked and dehulled. The seeds were polished using a modified non-contaminating Kett Mill with a milling time of 2 min 30 sec as previously described^13^. Polished grain samples were sent to Waite Analytical Services, University of Adelaide, Australia, for metal quantification. Samples were digested using nitric/perchloric acid on a programmable digestion system in open glass tubes^52^. Iron, zinc, and other metal concentrations were determined using inductively coupled plasma-atomic emission spectrometry (SPECTRO CIROS Radial).

### High-Cd soil experiment

Seeds of selected high-Fe transgenic events (IR64-NASFer-274 and −234), as well as wild-type control seeds of IR64 and a bulk of nulls, were germinated in petri dishes. Young seedlings were transferred into soil for 14 days to establish normal plant growth. Later, five plants per event were transferred into pots containing high-Cd soil from two locations in the Philippines, Santa Rosa in the province of Laguna (Cd availability as determined by the Analytical Service Laboratory at IRRI was 0.1038 ± 0.002881 mg kg^−1^) and San Leonardo in the province of Nueva Ecija (Cd availability was 0.246 ± 0.005477 mg kg^−1^). Completely randomized designs with three replications were employed. The plants were grown in the glasshouse under flooded conditions. Mature seeds were harvested, polished, and submitted to Waite Analytical Services, University of Adelaide, for digestion using nitric/perchloric acid on a programmable digestion system in open glass tubes^52^, and cadmium concentration was determined using ICP-MS at Flinders University.

### Grain quality evaluation

Seed samples of non-transformed IR64, homozygous transgenic plants, and the null of NASFer-274 were evaluated for six seed quality traits, including physical traits, amylose content, protein content, and gel consistency in IRRI’s Grain Quality and Nutrition Center Laboratory.

### *In vitro* iron bioavailability assessment

An *in vitro* digestion/Caco-2 cell culture model was used to assess Fe-bioavailability^48^,^49^. The rice samples were subjected to simulated gastric and intestinal digestion. Briefly, intestinal digestion is carried out in cylindrical inserts closed on the bottom by a semipermeable membrane and placed in wells containing Caco-2 cell monolayers bathed in culture medium. The upper chamber was formed by fitting the bottom of a Transwell insert ring (Corning) with a 15000 Da molecular weight cut-off (MWCO) membrane (Spectra/Por 2.1, Spectrum Medical, Gardena, CA). The dialysis membrane was held in place using a silicone ring (Web Seal, Rochester, NY). Iron uptake by the Caco-2 cell monolayers was assessed as previously described and by measuring ferritin concentrations in the cells^48^,^49^. The cells were maintained in Dulbecco’s modified Eagle medium plus 1% antibiotic/antimycotic solution, 25 mmol/L HEPES, and 10% fetal bovine serum. Forty-eight hours prior to the experiment, the growth medium was removed from the culture wells, the cell layer was washed, and the growth medium was replaced with minimum essential medium (MEM) at pH 7.0. The MEM was supplemented with 10 mmol/L PIPES, 1% antibiotic/antimycotic solution, 4 mg/L hydrocortisone, 5 mg/L insulin, 5 μg/L selenium, 34 μg/L triiodothyronine, and 20 μg/L epidermal growth factor. This enriched MEM contained less than 80 μg Fe/L. All ingredients and supplements for cell culture media were obtained from GIBCO (Rockville, MD). The cells were used in the Fe uptake experiment at 13 days post-seeding. In these conditions, the amount of cell protein measured in each well was highly consistent between wells. On the experiment day, 1.5 mL of the digested sample was added to the inserts’ upper chamber and incubated for 2 h. Then, the inserts were removed and 1 mL of MEM was added. Cell cultures were incubated for 22 h at 37 °C.

### Harvesting of Caco-2 cells for ferritin analysis

The protocols for ferritin and total protein content analyses were described previously^48^. Briefly, growth medium was removed from the culture well by aspiration and the cells were washed twice with a solution containing 140 mmol/L NaCl, 5 mmol/L KCl, and 10 mmol/L PIPES at pH 7.0. The cells were harvested by adding an aliquot of deionized water and placing them in a sonicator (Lab-Line Instruments, Melrose Park, IL). The ferritin and total protein concentrations were determined on an aliquot of the harvested cell suspension with a one-stage sandwich immunoradiometric assay (FER-IRON II Ferritin Assay, Ramco Laboratories, Houston, TX) and a colorimetric assay (Bio-Rad DC Protein Assay, Bio-Rad, Hercules, CA), respectively. Caco-2 cells synthesize ferritin in response to increases in intracellular Fe concentration. Therefore, we used the ratio of ferritin/total protein (expressed as ng ferritin/mg protein) as an index of cellular Fe uptake.

## Additional Information

**How to cite this article**: Trijatmiko, K. R. *et al*. Biofortified indica rice attains iron and zinc nutrition dietary targets in the field. *Sci. Rep.* **6**, 19792; doi: 10.1038/srep19792 (2016).

## Supplementary Material

Supplementary Information
