# Supplementary material for: Biofortified indica rice attains iron and zinc nutrition dietary targets in the field
Source: Sci Rep. 2016 Jan 25;6:19792. doi: 10.1038/srep19792 (PMC4726380; doi:10.1038/srep19792)
Supplement: Supplementary Information [file srep19792-s1.pdf]

Biofortified indica rice attains iron and zinc nutrition dietary targets in the field

Author names: Kurniawan R. Trijatmiko[a,j], Conrado Dueñas Jr.[a], Nikolaos Tsakirpaloglou[a], Lina Torrizo[a], Felichi Mae Arines[a], Cheryl Adeva[a], Jeanette Balindong[a], Norman Oliva[a], Maria V. Sapasap[a], Jaime Borrero[b], Jessica Rey[a], Perigio Francisco[a], Andy Nelson[c,d], Hiromi Nakanishi[e], Enzo Lombi[f], Elad Tako[g], Raymond P. Glahn[g], James Stangoulis[h], Prabhjit Chadha-Mohanty[a], Alexander A. T. Johnson[i], Joe Tohme[b], Gerard Barry[a], Inez H. Slamet-Loedin[a,k,]*

Author affiliations:

[a]Plant Breeding, Genetics, and Biotechnology Division, International Rice Research Institute, DAPO Box 7777, Metro Manila, Philippines
[b]Centro Internacional de Agricultura Tropical, Cali, Colombia
[c]Social Sciences Division, International Rice Research Institute, DAPO Box 7777, Metro Manila, Philippines
[d]Faculty of Geo-Information and Earth Observation (ITC), University of Twente, Enschede 7500 AE, The Netherlands
[e]Department of Global Agricultural Sciences, Graduate School of Agricultural and Life Sciences, The University of Tokyo, 1-1-1 Yayoi, Bunkyo-ku, Tokyo 113-8657 Japan
[f]Centre for Environmental Risk Assessment and Remediation, University of South Australia, Australia
[g]United States Department of Agriculture-Agricultural Research Service, Robert W. Holley Center for Agriculture and Health, Cornell University, New York
[h]School of Biological Sciences, Flinders University of South Australia, Adelaide, Australia
[i]School of Botany, The University of Melbourne, Victoria 3010, Australia
[j]Indonesian Center for Agricultural Biotechnology and Genetic Resources Research and Development, Bogor 16111, Indonesia
[k]Research Center for Biotechnology, Indonesian Institute of Sciences, Cibinong 16911, Indonesia

*To whom correspondence should be addressed. E-mail: i.slamet-loedin@irri.org

Supplementary Figure S1 Prevalence of micronutrient deficiency, high rice consumption and poverty; an unequivocal overlap is observed among all these areas. (a) Anaemia prevalence in children by country based on haemoglobin concentration[2]. Data are for 2011. **(b)** Annual per capita consumption of milled rice by country. Data from FAOSTAT Food Supply Data Online Database and USDA Production Supply and Distribution Online Database. Data are for 2011 (FAOSTAT) and 2013 (USDA). **(c)** Percentage of the population living on less than $1.25 a day at 2005 international prices. Data from World Bank, Development Research Group. Data are for the most recent year of primary household survey data obtained from government statistical agencies and World Bank country departments. All maps were generated using the ArcGIS 10.0 ((c) ESRI) GIS software.

a

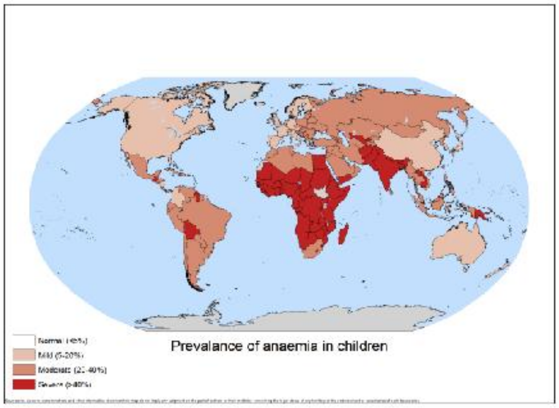

b

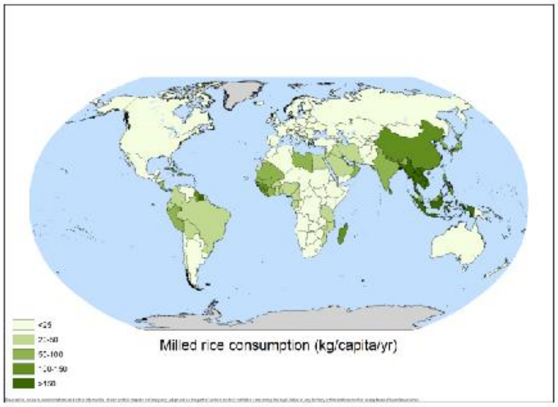

c

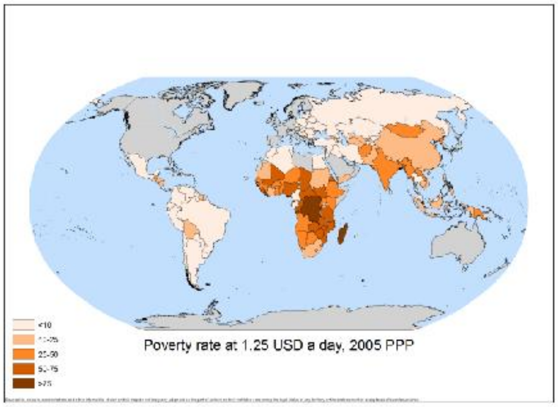

Supplementary Figure S2 PCR-based assay for zygosity test of the lead events. **(a** and **c)** Schematic diagram of PCR-based assay using three oligonucleotides, P1, P2, and P3, for zygosity test for the -234 **(a)** or -274 **(c)** insertion. **(b** and **d)** Zygosity test on genomic DNA from a non-transformed plant (WT control), plants hemizygous for the -234 **(b)** or -274 **(d)** insertion, and plants homozygous for the -234 **(b)** or -274 **(d)** insertion or null control. One kb plus DNA ladder was used as size standard.

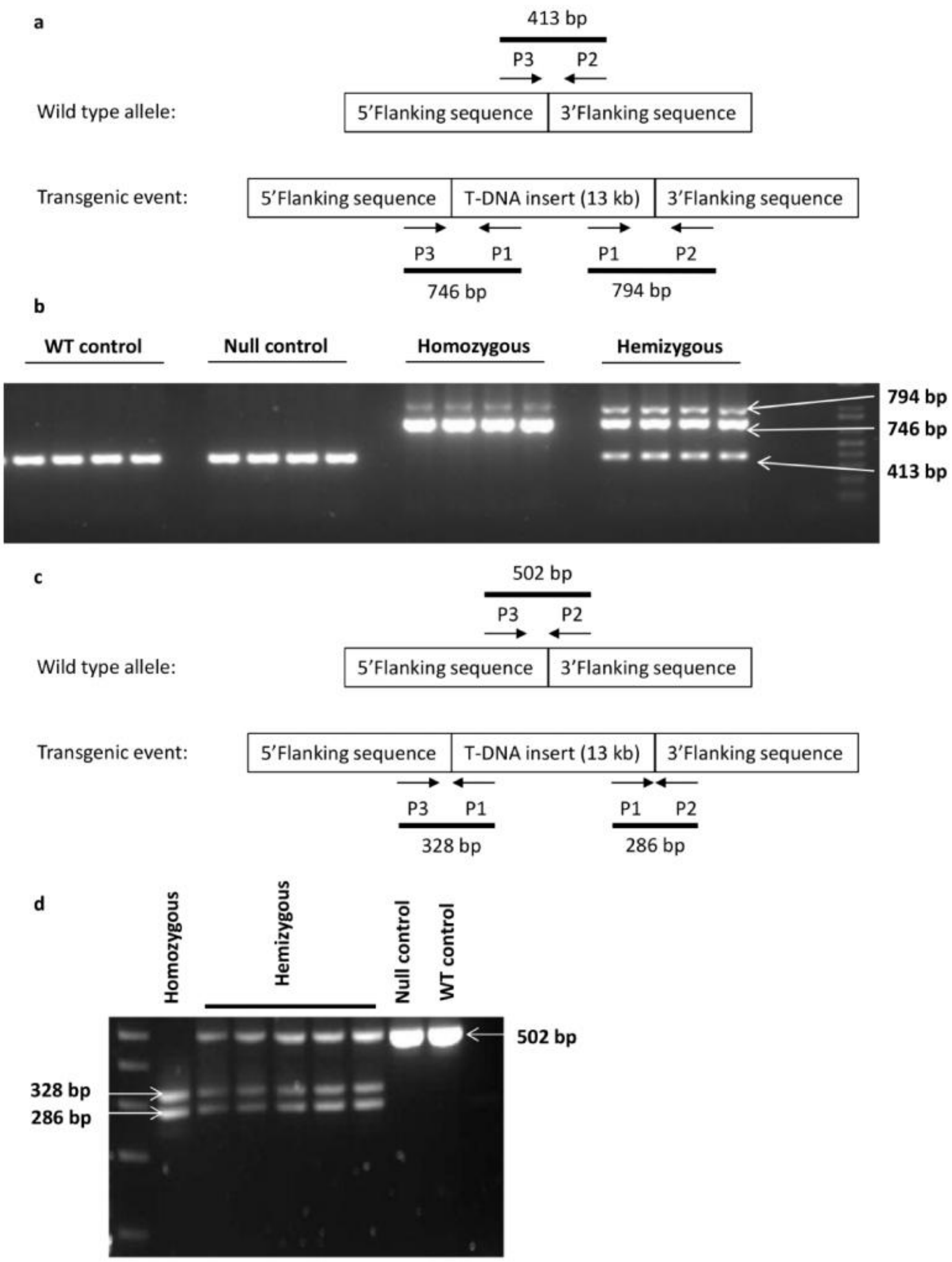

Supplementary Figure S3 Relative quantification of endogenous *OsNAAT1* (**a**) and *OsDMAS1* (**b**). T_2 homozygous plants were cultivated in pots under screenhouse conditions. Total RNA was extracted from vegetative-stage leaves from each line (n = 2). Bars represent the means ± s.d. of two biological replicates, each with three technical replicates of real-time RT-PCR.

a

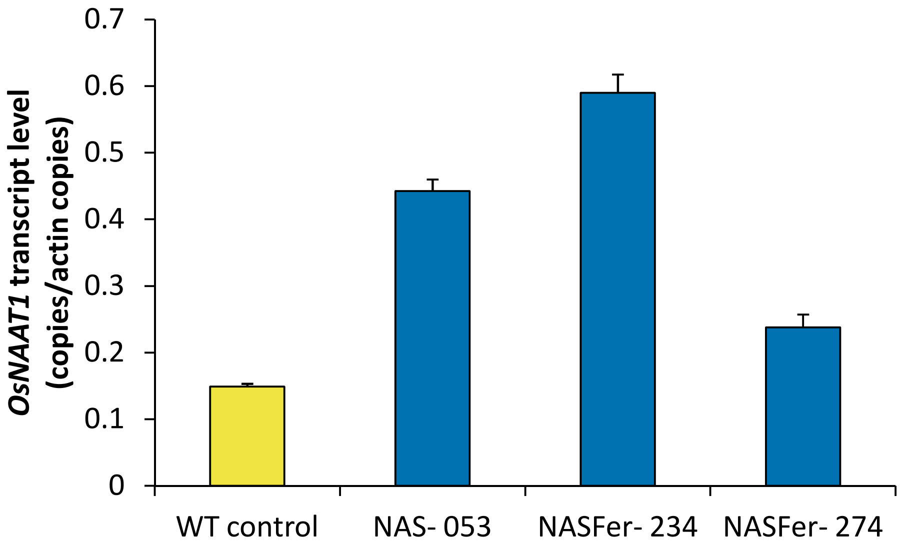

b

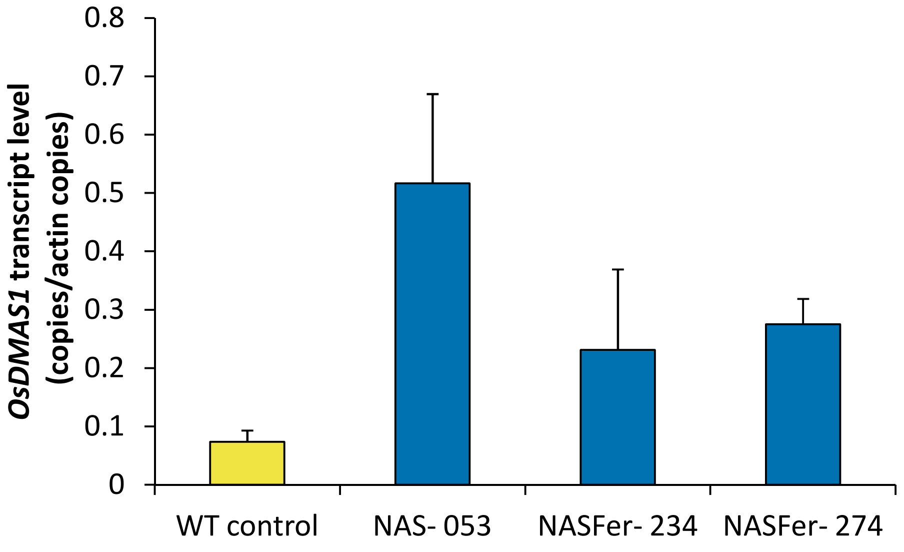

Supplementary Figure S4 Field trials for evaluation of target trait and agronomic characters of lead events at IRRI Philippines. A represents WT control, B represents NASFer-274, C represents Null-274.

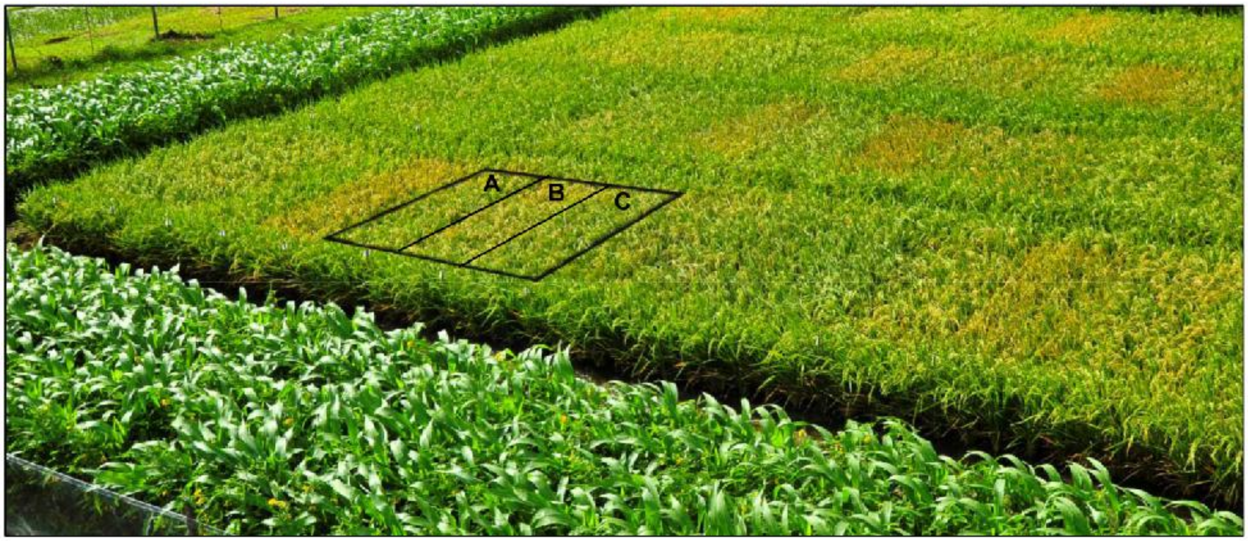

Supplementary Figure S5 Microscopy images of polished rice showing the absence of bran.

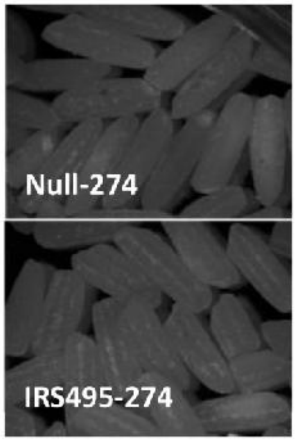

Supplementary Figure S6 Flanking sequences and position of T-DNA integration in event NASFer-234. **(a)** Rice genome sequence upstream of the T-DNA. **(b)** Rice genome sequence downstream of the T-DNA. Green and black sections indicate rice genome and T-DNA insert sequences, respectively. Alignment to the draft genome sequence of IR64 was done at a specialized deployment of Galaxy at the International Rice Research Institute (http://175.41.147.71:8080/). Left Border repeat was deleted during T-DNA integration in both cases **(c)** Position of T-DNA integration in the second exon of a gene encoding hypothetical protein on chromosome 6. Manual annotation was done using the gene prediction program FGENESH with the Oryza_indica rice training set (www.softberry.com). Brown and blue sections indicate exon and intron, respectively. The section with green highlight represents nucleotides deleted during T-DNA integration.

a

```
                                       IR64 draft1:scaffold_745 position 13,264↓
AGCGAGCGAGAGAGGAATTAAGGCAGCGGGATGGCGCGATTTGTGGGGATGCGGCGGCGGTGGCGACGGCAAGGATC
     filler DNA                                   Left Border region
TGGATTTTTTAAACAAATTGACGCATTGCGGACGTTTTTAATGTACTGAATTAACGCCGAATTAATTCGGGGGATCT

GGATTTTAGTACTGGATTTTGGTTTTAGGAATTAGAAATTTTATTGATAGAAGTATTTTACAAATACAAATACATAC

TAAGGGTTTCTTATATGCTCAACACATGAGCGAAACCCTATAGGAACCCTAATTCCCTTATCTGGGAACTACTCACA

CATTATTATGGAGAAACTCGAGCTTGTCGATCGACAGATCCCGGTCGGCATCTACTCTATTTCTTTGCCCTCGGACG

AGTGCTGGGGCGTCGGTTTCCACTATCGGCGAGTACTTCTACACAGCCATCGGTCCAGACGGCCGCGCTTCTGCGGG

CGATTTGTGTACGCCCGACAGTCCCGGCTCCGGATCGGACGATTGCGTCGCATCGACCCTGCGCCCAAGCTGCATCA

TCGAAATTGCCGTCAACCAAGCTCTGATAGAGTTGGTCAAGACCAATGCGGAGCATATACGCCCGGAGTCGTGGCGA

TCCTGCAAGCTCCGGATGCCTCCGCTCGAAGTAGCGCGTCTGCTGCTCCATACAAGCCAACCACGGCCTCCAGAAGA

AGATGTTGGCGACCTCGTAT
```

b

```
ATACGAGGTCGCCAACATCTTCTTCTGGAGGCCGTGGTTGGCTTGTATGGAGCAGCAGACGCGCTACTTCGAGCGGA

GGCATCCGGAGCTTGCAGGATCGCCACGACTCCGGGCGTATATGCTCCGCATTGGTCTTGACCAACTCTATCAGAGC

TTGGTTGACGGCAATTTCGATGATGCAGCTTGGGCGCAGGGTCGATGCGACGCAATCGTCCGATCCGGAGCCGGGAC

TGTCGGGCGTACACAAATCGCCCGCAGAAGCGCGGCCGTCTGGACCGATGGCTGTGTAGAAGTACTCGCCGATAGTG

GAAACCGACGCCCCAGCACTCGTCCGAGGGCAAAGAAATAGAGTAGATGCCGACCGGGATCTGTCGATCGACAAGCT

CGAGTTTCTCCATAATAATGTGTGAGTAGTTCCCAGATAAGGGAATTAGGGTTCCTATAGGGTTTCGCTCATGTGTT

GAGCATATAAGAAACCCTTAGTATGTATTTGTATTTGTAAAATACTTCTATCAATAAAATTTCTAATTCCTAAAACC
                                        Left Border region
AAAATCCAGTACTAAAATCCAGATCCCCCGAATTAATTCGGCGTTAATTCAGTACATTAAAAACGTCCGCAATGTGT
    filler DNA ↓IR64 draft1:scaffold_745 position 13,307
TATTAAGTTGTGAGAGAATGCGCGTTCTGAGAGAGGAGGGGAGAGCCGGAGAGGTGCGTGCGGATGGGACGGGAGAG

AGGAAACGTGGGACTTTCTGAGGCGTTGGATGAGATGATTCTGGATCGTCGAATTTTTTTTTTTTTTTAGAATTACA

CGGTACAACGCAGACACTCACAACGCACGCGCACTCACCCCTATGAACACACGCATGCAA
```

c

Start codon
ATGGGAGCGAATTTTTCAGGATTTAACGGCGTTGTGCTTTCAGTGGTAGGCGATGTACCCGTCGACAGCGAGGCGTC

TGTGGTGATTTCGTCAATCTCTCCAGAATTTGTCGGCCCAGTCTTCAAAGATGCTCATAGGGGTAGGGTTTGCATGC

GTGTGTTCATAGGGGTGAGTGCGCGTGCGTTGTGAGTGTCTGCGTTGTACCGTGTAATTCTAAAAAAAAAAAAAAT

TCGACGATCCAGAATCATCTCATCCAACGCCTCAGAAAGTCCCACGTTTCCTCTCTCCCGTCCCATCCGCACGCACC

í ***T-DNA integration***
TCTCCGGCTCTCCCCTCCTCTCTCAGAACGCGCATTC~~CCTCATCTCTCGGTCGCTCCCCTCGACCGCCGCCGCATCC~~

í
~~AC~~GATCCTTGCCGTCGCCACCGCCGCCGCATCCCCACAAATCGCGCCATCCCGCTGCCTTAATTCCTCTCTCGCTCG

CTCCCCCTCAACCGCTGCCGCTGCCGCTGCCGCTGCCGCCGCCTCCGCATCCCTAATCCACTCCGCGCCGTCTCCTA

CGCCGGTCGAGATCTCCGCCATCGTCGCCACGCACAGCCACCTGAGCCTCTCTTCCCGTCCCCTTGTGCCCTTCCAT

GTCCTCTTCTTTCTCCCTCTCTCTCTATCTTGATCGAACCCTAGGTCCTCGCGGTGTGGCTCTCCCGGAGCAAGCGA

GCCAGCATGGCAGAGTCGATCCGCGTCATCATGGAGGTGGGCGCCGACGGCGTCATGCTATGCTCGTTACCCTCTGC

AACCCGCCGGTCAATGTGCTGCATCCCATTAGTAAACCCTCCTCGCTTTCTTAATCTCGATTTTATTTTTTTGCCCA

TCTCTTGGTGTTGGGGTTTTTGGTGTTGCTGGTCACTGATTCCATGCACGGGTTTGGATTTTGCTCCCCATGCGTCG

TGCAGTCATCCAGGGGCTCAAGGAGAAGTACGCGGAGGCATGGATCGCGACGAAGTCAAGGCAATCGTGCTCACCGG

TGAGGCCTCCCGATAATTGTTTCACCCCCCCTCCCTACGAAAAATTGATCAGAGAATTCATTACCTATACACAACTG

AAATATCTGATTTGTTTATTGCGGCAGCCGTCGAATTTGCGCTCATTTTGTACTGATAGATTGCTGTACGGGTTGAC

GTAAGATGAGCAGCATTTTTGTTTGTTTTGCTGGTTTTTCGAGTGGTTGTGGAGTAGTCGCTGCTATCCAATCATTA

TGTGCTGATCTCAGTCAACTAGGTTAATGAGTGGGCTGCTCCTGCTGTACAAGATTTGCTTTGCTGGTGGTCTAGAA

CTGTAGTGGAGCTGCTCAACTGTAAATGGCAAATGTTGGATGCTAATCCAGTTATCTCACTAAAGTAGAAACTACAG

CAATGGCAAGTTATATATAAACCTTGATCAGAAAGGAACACAATTTTGTACAGGTCCCCAAGGAGATCTGTTTTCTT

CAAACATTCTTTCCTATTAATGGGTAGGAAATAGCTTTTCTGGTAACTTTTGGTTGAAATGTGCCGTTGTACATTTT

AGGAATGCAAGAGTGAGAAAGGGGATTGTAAATTTTAGTTTATCATACTTGTTGTTTTTCTTCTAAATTATGTTCAA

TCCTTGCTAGATACTGGAGGCAAATTTTGTGGAGGCTTTAATATCAATGTGTTCACAGAAGCTCACAAGACTGGTTA

ATGGTCCTGTCATCGACTCGTGGCCTCTGTCCCCCGACACCTGCCCGAGTCGCCGCCTCTGTCCCTCAACACCACCC

Stop codon
AGACCTCCACCGACTCCATCCGTGTCTGCATCATCGGCTGCAATC**TAG**

Supplementary Figure S7 Flanking sequences and position of T-DNA integration in event NASFer-274. **(a)** Rice genome sequence upstream of the T-DNA. **(b)** Rice genome sequence downstream of the T-DNA. Green and black sections indicate rice genome and T-DNA insert sequences, respectively. **(c)** Position of T-DNA integration in the 3'UTR of LOC_Os03g55720. Brown, blue, and black sections indicate coding sequence, UTR, and intergenic region, respectively. The section with blue highlight represents nucleotides deleted during T-DNA integration.

a

Rice chromosome 3 position 32,576,511↓
TCGATTAAAATTTGCTTTCGTTTTGTACCCATGACGATTGGTGATATATTGTGGTGTAAACAAATTGACGCTTAGAC
Left Border
AACTTAATAACACATTGCGGACGTTTTTAATGTACTGAATTAACGCCGAATTAATTCGGGGGATCTGGATTTTAGTA

CTG

b

CTGGCAAACTGTGATGGACGACACCGTCAGTGCGTCCGTCGCGCAGGCTCTCGATGAGCTGATGCTTTGGGCCGAGG

ACTGCCCCGAAGTCCGGCACCTCGTGCACGCGGATTTCGGCTCCAACAATGTCCTGACGGACAATGGCCGCATAACA

GCGGTCATTGACTGGAGCGAGGCGATGTTCGGGGATTCCCAATACGAGGTCGCCAACATCTTCTTCTGGAGGCCGTG

GTTGGCTTGTATGGAGCAGCAGACGCGCTACTTCGAGCGGAGGCATCCGGAGCTTGCAGGATCGCCACGACTCCGGG

CGTATATGCTCCGCATTGGTCTTGACCAACTCTATCAGAGCTTGGTTGACGGCAATTTCGATGATGCAGCTTGGGCG

CAGGGTCGATGCGACGCAATCGTCCGATCCGGAGCCGGGACTGTCGGGCGTACACAAATCGCCCGCAGAAGCGCGGC

CGTCTGGACCGATGGCTGTGTAGAAGTACTCGCCGATAGTGGAAACCGACGCCCCAGCACTCGTCCGAGGGCAAAGA

AATAGAGTAGATGCCGACCGGGATCTGTCGATCGACAAGCTCGAGTTTCTCCATAATAATGTGTGAGTAGTTCCCAG

ATAAGGGAATTAGGGTTCCTATAGGGTTTCGCTCATGTGTTGAGCATATAAGAAACCCTTAGTATGTATTTGTATTT

GTAAAATACTTCTATCAATAAAATTTCTAATTCCTAAAACCAAAATCCAGTACTAAAATCCAGATCCCCCGAATTAA
Left Border
TTCGGCGTTAATTCAGTACATTAAAAACGTCCGCAATGTGTTATTAAGTTGTCTAAGCGTCAATTTGTTTACACCAC
filler DNA ↓*Rice chromosome 3 position 32,576,534*
AATATACATTAAAAACGGTTAGCGGTTTTGGAGGCAGAACCTGCAAATGCAATTGGTTTTTCAGCTGTACACATTGC

GGACTTGCTACGGTCTGTCCAATTCTTCTTCCAGAGTTTACTTCGGTTTTGAAACATTCAAAAACCAGAATGATCA

CATCATCACCATCATTCATCAGCACTGAATCATGTTTAGTGTACTCACTTTCCCATAAACTGATGACAAAACTGAAT

GTTATAGGCTTTCATTTTAGATGATGCCATGCGGTTGACAGAAGAA

c

→ *5’UTR of LOC_Os03g55720*

ATCCATTCCGCCGGCGCCTTATCCTACCCTCATCTCCCGTACAAAACCCACTCCCTCCCTCCCGCTCCCAACCTGAC

CCACTCCAACCCCCCCTGCGCCATTGCCGGCCACACTCCTCTTCCTCCTCCATCGCCAAGAACCACCACGAGGTAGG

CAAACCACCCTCATGGAGATGGATTTCTGTCGTTCTGAGCTGCAACATTGCTTGATCTCATCGTTGGTTTTCTTGCA

← í*Start codon of LOC_Os03g55720*

GCA**ATG**GCGTCGCTGTCCATGGCCACAACGCTGCCGTCGCTGGCCGGCGCCGCACCGGCGGCGAGGAAGAGGAGCGG

CGTGACGTACGTGGAGGGGATGAACGCGTACAGCGGGCTCAAGGCGCTCAACAAGGTGACCCTGCTCGGCGTGCGCA

AGACCGCCGACTACTCGTTCGCCAAGGTGGTGGCCAAGCTGAGCCCCGCCGGCGGGAAGAGCCGCGGCGGCGCGTTC

GGGGCGCAGTGCAACGCCGCCGGTGAGATATTCAGGATCGCCGTCATCATGAACGGGCTCGTGCTCGTCGGCGTCGC

*Stop codon of LOC_Os03g55720*í → *3’UTR of LOC_Os03g55720*

CGTCGGCTTCGTGCTGCTCCGGGTGGAGGCGGCCGTCGAGGAGTCGGAG**TAG**TGGACCCCGATCGAGCCATAGCAGG

CAGGGCCCAGCCAGCCATGGCTGGTTGGCCCCGATAAGCCGTGATATATAACTGTGATGTATGTTTCGATTAAAATT

í *T-DNA integration* í

TGCTTTCGTTTTGTACCCATGACGATTGGT~~GAATACCCTTGGAACGAAACAC~~GTTTTGGAGGCAGAACCTGCAAATG

←

CAATTGGTTTTTCAGCTGTACACATTGCGGACTTGCTACGGTCTGTCCAATTCTTCTTCCAGAGTTTACTTCGGTTT

→ *3’UTR of LOC_Os03g55730*

TGAAACATTCAAAAAACCAGAATGATCACATCATCACCATCATTCATCAGCACTGAATCATGTTTAGTGTACTCACT

TTCCCATAAACTGATGACAAAACTGAATGTTATAGGCTTTCATTTTAGATGATGCCATGCGGTTGACAGAAGAAGCA

CAGCAGGGGCAAAATATACTTTTAGTTCAATGCCTATAAGAGCTGTTAACAATGGCAACTCACTGGAGCATGTGTTT

CTCCACTAAAACTTACAGAAAAAGGATTCTAAATAATAAGTTCTATGATCGAACCCCCAGAAAAGATAGTGTCAAGG

CTGAAAAGGCGCATCCTAATTCTGTGGAAGTACAACAGGAGCCATCTCAGGAGCGTGAATCTCCGGCAATTTGCAAA

← í*Stop codon of LOC_Os03g55730*

CTCATTCC**TTA**CATTGGTCTCTGACAAGCTGAGAACAGCTGCCAGCTGATGAAAGCCATGACCAGTGAGGAGAACAG

CTCGAAGCCAACCACCTTTGGAGTCTGCCACTCTAATGATCTCCTCAAGTAACTGCATGTTTAGCACAGTGAAGAAG

Supplementary Figure S8 Histochemical X-Gluc staining of longitudinal sections of pr*GluA2*::*GUS* transgenic rice seeds at 7, 12, 17, and 30 days after flowering (DAF).

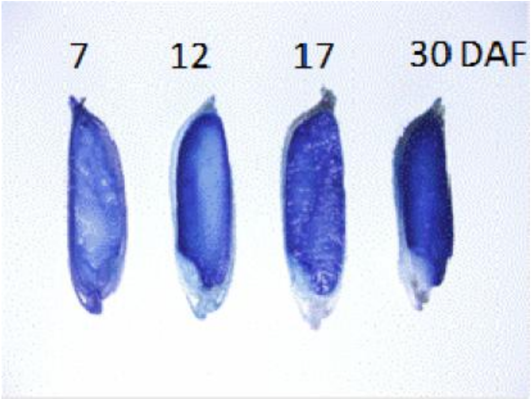

Supplementary Table S1 Overview on generated transgenic T_0 lines transformed with seven constructs and color image analysis for Fe in rice grains for each construct.

Construct ID	Description of the cassette	No. of events	Percentage of events (%)		
			High staining[a]	Medium staining	Low staining
IRS429	prUbi::OsNAS2::tNOS	254	2	17	81
IRS433 (NAS)	pr35S::OsNAS2::tNOS	158	**4**	11	85
IRS461	prGluB1:: OsNAS2::tNOS	220	2	47	51
IRS489	prGluB1::SFerH-1::tNOS	228	0	38	62
IRS491 (Fer)	prGluA2:: SFerH-1::tNOS	188	**32**	26	42
IRS493	pr35S::OsNAS2::tNOS+ prGluB1::SFerH-1::tNOS	295	13	81	6
IRS495 (NASFer)	pr35S::OsNAS2::tNOS+ prGluA2:: SFerH-1::tNOS	346	**89**	11	0
	**Total**	**1,689**	142	231	327

[a]Based on mean gray value (relative to that of the untransformed IR64 control) of scanned Prussian blue-stained seeds analyzed with ImageJ (see Methods). Low staining: 120-130%, Medium staining: 131-140%, High staining: >140%.

Supplementary Table S2 Fe concentration in polished seeds of selected events from 3 selected constructs.

Construct	No. of events	No. of selected events	No. of single-insert events	Selected event designation	Fe concentration of homozygous polished grains (ug g^{-1} DW)	Beyond T-DNA border transfer
IRS495 (NASFer)	346	33	12	NASFer-199	8.7	Yes
				NASFer-234	11.7	No
				NASFer-238	10.0	Yes
				NASFer-274	15.0	No
				NASFer-296	5.5	No
IRS491 (Fer)	178	26	13	Fer-85	8.8	No
				Fer-98	8.0	No
				Fer-113	7.4	No
				Fer-137	8.3	No
				Fer-168	7.1	No
IRS433 (NAS)	158	23	1	NAS-53	8.2	No

Supplementary Table S3 Grain quality of non-transformed IR64, event NASFer-274, and null from event NASFer-274 grown in the confined field trial.

Line	Grain quality parameters					
	Chalkiness (%)	Length (mm)	Width (mm)	Amylose content (%)	Gel consistency (mm)	Protein content (%)
NASFer-274	1.3 ± 0.6[a]	6.23 ± 0.01	1.92 ± 0.01	27.7 ± 0.3	100 ± 0.0	10.4 ± 0.2
Null-274	0.7 ± 0.6	6.30 ± 0.02	1.92 ± 0.01	27.0 ± 0.5	89 ± 6.1	11.0 ± 0.3
IR64-WT	3.7 ± 1.2	6.33 ± 0.01	1.89 ± 0.02	23.4 ± 0.3	100 ± 0.0	10.5 ± 0.2

[a]Values are means ± s.d., n = 3

Supplementary Table S4 Metal concentrations of T_3 polished grains of events NASFer-234 and NASFer-274 and their nulls grown in the confined field trials.

Location	Event	Fe ($\mu g\ g^{-1}$)	Zn ($\mu g\ g^{-1}$)	P ($\mu g\ g^{-1}$)	S ($\mu g\ g^{-1}$)	Cd ($\mu g\ g^{-1}$)	Pb ($\mu g\ g^{-1}$)	As ($\mu g\ g^{-1}$)
CIAT-Colombia	NASFer-234	14.7 ± 0.5 [a]	53.8 ± 5.4	1803.3 ± 136.5	1453.3 ± 80.8	< 0.1[b]	< 1[c]	< 1[d]
	Null-234	2.9 ± 0.2	16.5 ± 1.4	1460.0 ± 110.0	1256.7 ± 56.9	< 0.1	< 1	< 1
	NASFer-274	15.0 ± 0.4	45.7 ± 1.8	1505.0 ± 21.2	1365.0 ± 49.5	< 0.1	< 1	< 1
	Null-274	4.2 ± 0.4	16.6 ± 1.2	1735.0 ± 106.1	1380.0 ± 56.6	< 0.1	< 1	< 1
	IR64-WT	2.5 ± 0.0	14.2 ± 0.4	1326.7 ± 20.8	1153.3 ± 20.8	< 0.1	< 1	< 1
IRRI-Philippines	NASFer-234	13.2 ± 0.9	44.7 ± 2.0	1575.0 ± 149.3	1442.5 ± 41.1	< 0.1	< 1	< 1
	Null-234	2.9 ± 0.4	17.3 ± 1.4	1500.0 ± 112.0	1295.0 ± 40.4	< 0.1	< 1	< 1
	NASFer-274	14.6 ± 1.2	49.0 ± 2.3	1725.0 ± 167.0	1455.0 ± 85.4	< 0.1	< 1	< 1
	Null-274	3.4 ± 0.4	19.2 ± 1.2	1805.0 ± 67.6	1377.5 ± 134.5	< 0.1	< 1	< 1
	IR64-WT	2.6 ± 0.2	16.2 ± 1.9	1327.5 ± 80.6	1167.5 ± 116.2	< 0.1	< 1	< 1

[a]Values are means ± s.d., n = 3
[b]Detection limit for Cd concentration using ICP is 0.1 $\mu g\ g^{-1}$
[c]Detection limit for Pb concentration using ICP is 1 $\mu g\ g^{-1}$
[d]Detection limit for As concentration using ICP is 1 $\mu g\ g^{-1}$

Supplementary Table S5 Ferritin concentrations in Caco-2 cells exposed to samples of rice digests.

Sample tested	Caco-2 cell ferritin (ng/mg of total protein[a])	Increased ferritin amount (ng/mg of total protein[b])
Cell baseline	1.85 ± 0.18	0
NASFer-274	6.14 ± 1.00	4.29
NASFer-234	8.77 ± 1.13	6.92
IR64 wild type	2.54 ± 0.49	0.69
NASFer-274+AA[c]	18.76 ± 2.00	18.66
NASFer-234+AA	26.27 ± 2.41	26.17
IR64 wild type+AA	4.44 ± 0.15	4.34

[a]Values are means ± s.d., n = 3
[b]Values are normalized to the cell baseline
[c]Ascorbic acid (AA) is added to the sample

Supplementary Table S6 Chemical properties of soil at IRRI CFT.

Chemical property	
pH	5.65 ± 0.071[a]
Organic C %	1.22 ± 0.106
Available K (meq/100g)	0.83 ± 0.021
Available P-B (ppm)	9.10 ± 0.424
Available P-O (ppm)	15.50 ± 0.707
Cation exchange capacity (meq/100g)	39.20 ± 1.131
Active Fe (%)	1.68 ± 0.262
Available Zn (ppm)	0.45 ± 0.099
Clay H %	57 ± 0
Sand H %	10 ± 0
Silt H %	33 ± 0

[a]Values are means ± s.d., n = 2

Supplementary Table S7 Chemical properties of soil at CIAT CFT.

Chemical property	
pH	7.85 ± 0.0371[a]
Organic matter (g/kg)	17.99 ± 1.279
P (mg/kg)	299.80 ± 59.419
K (cmol/kg)	0.84 ± 0.063
Ca (cmol/kg)	17.60 ± 1.436
Mg (cmol/kg)	6.29 ± 0.900
Na (cmol/kg)	0.31 ± 0.010
Cation exchange capacity (cmol/kg)	18.06 ± 1.552
S (mg/kg)	22.58 ± 12.368
B (mg/kg)	0.60 ± 0.157
Cu (mg/kg)	0.94 ± 0.645
Fe (mg/kg)	4.84 ± 3.840
Mn (mg/kg)	87.39 ± 34.530
Zn (mg/kg)	6.22 ± 3.293

[a]Values are means ± s.d., n = 5

Supplementary Table S8 Primers for confirmation of T-DNA inverted repeat configuration in event NASFer-274.

No	Primer name	Sequence (5′–3′)	Amplicon size (bp)
1	prGluA2-F	GCAAGCTTGTTAATCATGGTGTAGGCAACC	4,664
	274-upstream flanking	TTCTTCTGTCAACCGCATGG	
2	SferH1-RT-F	CTTGCTGTTCCAACTGCTCC	3,517
	274-upstream flanking	TTCTTCTGTCAACCGCATGG	
3	tNOS-F	GATCGTTCAAACATTTGGCA	2,877
	274-upstream flanking	TTCTTCTGTCAACCGCATGG	
4	prGluA2-F	GCAAGCTTGTTAATCATGGTGTAGGCAACC	4,622
	274-downstream flanking	GGATCGCCGTCATCATGAAC	
5	SferH1-RT-F	CTTGCTGTTCCAACTGCTCC	3,475
	274-downstream flanking	GGATCGCCGTCATCATGAAC	
6	tNOS-F	GATCGTTCAAACATTTGGCA	2,835
	274-downstream flanking	GGATCGCCGTCATCATGAAC	

Supplementary Methods

Generation of plant transformation vectors and transgenic rice. Fragments encompassing the full-length coding regions were amplified (using KAPA Hifi DNA Polymerase Hotstart) from young soybean leaf cDNA (for *SferH-1*) or rice genomic DNA (*OsNAS2*, LOC_Os03g19420). Soybean cv. PHI29924 and rice cv. Nipponbare were used. Oligonucleotides SferH1-F (5′-gcggatccCACAAATCTTAGCCGCCATT-3′) and SferH1-R (5′-gcggtaccCCAGAATTTCAGAAAAGACCAAATG-3′) were used to amplify *SferH-1*, whereas oligonucleotides OsNAS2-F: 5′-GAGAGAggatccATGGAGGCTCAGAACCAAGA-3′ and OsNAS2-R:5′-GAGAGAggtaccTCAGACGGATAGCCTCTTGG-3′[24] were used to amplify *OsNAS2*. Both pairs of oligonucleotides introduced *BamH*I and *Kpn*I restriction sites (indicated in lower case) to the amplified fragments at their 5′ and 3′, respectively. The oligonucleotides pr35S-F: 5′-GAGAGAaagcttCATGGAGTCAAAGATTCAAA-3′ and pr35S-R:5′-GAGAGAggatccAGTCCCCCGTGTTCTCTCCA-3′ were used to amplify a 538-bp fragment of the CaMV 35S promoter from pCAMBIA1301. The oligonucleotides prGluA2-F: 5′-GCaagcttGTTAATCATGGTGTAGGCAACC-3′ and prGluA2-R: 5′-GCggatccGTTGTTGTAGGACTAATGAACTGAATG-3′ were used to amplify a 839-bp GluA-2 promoter[31] from rice cv. Nipponbare genomic DNA. Both pairs of oligonucleotides introduced *Hind*III and *BamH*I restriction sites (indicated in lower case) to the amplified fragments at their 5′ and 3′, respectively. In all cases, fragments were introduced to the pCR4Blunt-TOPO vector as described by the manufacturer (Life Technologies) and subsequently sequenced from both sides. After digestion, fragments (promoter and gene) with appropriate compatible cohesive ends were ligated in between the *Hind*III and *Kpn*I sites of pCAMBIA1300int-tNOS (provided by

Emmanuel Guiderdoni, CIRAD, France) to create the plant transformation vector pCAMBIA1300int-pr35S-OsNAS2-tNOS (IRS433) and pCAMBIA1300int-prGluA2-SferH1-tNOS (IRS491). To generate the two-cassette construct, the oligonucleotides 35S-OsNAS2-HF (5′-GCaagcttCATGGAGTCAAAGATTCAAATAGA-3′) and 35S-OsNAS2-HR (5′-GCaagcttCCGATCTAGTAACATAGATGACACC-3′) were used to amplify the pr35S-OsNAS2-tNOS cassette from IRS433. The pair of oligonucleotides introduced the *Hind*III restriction site (indicated in lower case) to the amplified fragment at its 5′ and 3′, which was used to ligate the cassette to the *Hind*III site in IRS491 to create pCAMBIA1300int-pr35S-OsNAS2-tNOS-prGluA2-SferH1-tNOS (IRS495). For generating the promoter:GUS construct, the *GUS* gene was amplified from pCAMBIA-1301 (CAMBIA, Australia) and used to replace *SferH-1* gene in the IRS491, to create pCAMBIA1300int-prGluA2-GUS-tNOS. Transformation of indica rice cv. IR64 was performed using an immature embryo as previously described[50]. Transgenic plants were grown in a greenhouse. The presence of a transgene in the transformants was confirmed by PCR using the primer pair pr35S-F and pr35S-R for IRS433, GluA2-SferH1-F (5′-GAACAACACAATGCTGCGTC-3′) and GluA2-SferH1-R (5′-GCTCGCTCTTCTTAACCTCC-3′) for IRS491, and OsNAS2-GluA2-F (5′-CAAGTGCTGCAAGATGGAGG-3′) and OsNAS2-GluA2-R (5′-AGGCTCATCGAGGATACACG-3′) for IRS495.

Selection of construct based on color image analysis for Fe. A semi-quantitative analysis of Fe concentration[51] with brief modifications was performed on brown seeds for the mass screening of the generated transgenic events. Ten T_1 seeds from each T_0 transgenic line were dehusked to obtain brown seeds. The seeds were placed in a 24-well flat-bottom plate containing water and vacuum infiltrated. A 1-mm cross section was made in the middle part of each seed using a

ceramic knife. The cross sections were soaked in 2% HCl and 2% potassium hexacyanoferrate (II) trihydrate for 1 h. After six times washing with water, the staining was intensified with 0.3% (v/v) H_2O_2 in methanol. The cross sections were rinsed six times with water and kept in 70% ethanol. For embedding, the 70% ethanol was removed and a warm suspension of 0.75% agarose was added to the well. The sections were arranged close to each other before the agarose solidified. The plates were scanned using a color scanner (ApeosPort-IV C3370), and saved as a TIFF file with 600 dpi resolution. The TIFF file was converted into an HSB file and the intensities of the blue staining of each event were quantified using ImageJ software.

Selection of events from selected constructs using Fe staining on polished seeds. T_1 seeds from each T_0 transgenic event of IRS433, IRS491 and IRS495 were dehusked to obtain brown seeds. To polish the seeds, 30 brown seeds were placed into 2-mL tubes. The 2-mL tubes were subsequently placed in a Cryo-Block for 48 Microcentrifuge (SPEX SamplePrep) and shaken vigorously for 2 min at 1,200 strokes per min for 70 cycles using a 2000 Geno/Grinder (SPEX SamplePrep). Three polished seeds of each T_0 plant were cut transversely across the middle plane of the grain by a ceramic knife. Then, the samples were soaked in 2% HCl and 2% potassium hexacyanoferrate (II) trihydrate for 2 h. Stained samples were washed with distilled water and observed by stereoscopic microscope (SZX7 of Olympus Corp.).

T-DNA flanking sequence recovery. Genomic sequences flanking the T-DNA left border were cloned using inverse PCR or TAIL-PCR as previously described[58-60]. For inverse PCR, genomic DNA (1 μg) obtained from each event was digested with *Taq*I, self-ligated with T4 DNA ligase (Invitrogen), ethanol-precipitated, digested with *Apo*I, and then amplified by nested PCR with

the following primer sets: IPCR1-F (5′-CAGTACTAAAATCCAGATCCCCCGAAT-3′) and IPCR1-R (5′-TTATATGCTCAACACATGAGCGAAACC-3′); IPCR2-F (5′-ACGTCCGCAATGTGTTATTAAGTTGTC-3′) and IPCR2-R (5′-AACCCTAATTCCCTTATCTGGGAACTA-3′). These sets were used in the first and second rounds of PCR, respectively. For TAIL-PCR, genomic DNA (50 ng) obtained from each event was amplified using left-border-specific primer LB1 (5′-CAGTACTAAAATCCAGATCCCCCGAAT-3′) in combination with AD1, AD2, AD3, or AD4 primer[61] with thermal conditions for primary amplification as previously described[60]. Secondary amplification was performed as previously described[60] using 1 μL of the primary amplification product as a template with the nested left-border-specific primer LB2 (5′-ACGTCCGCAATGTGTTATTAAGTTGTC-3′) and the same AD primer. DNA of the non-transformed plant was used as a control. Transgenic event-specific amplification products were excised from the agarose, purified using GeneClean Kit II (QBiogene), cloned into pGEM-T Easy (Promega), and sequenced. Identification of the insert position in the rice genome was performed using a BlastN algorithm[55] at the National Center for Biotechnology Information (www.ncbi.nlm.nih.gov).

Amplification and sequencing for confirmation of T-DNA inverted repeat configuration in event IRS495-274. A primer that is complementary to a genetic element within T-DNA insert was paired with either primer that is complementary to the upstream flanking sequence or primer that is complementary to the downstream flanking sequence to amplify genomic DNA of a homozygous T_1 NASFer-274 line using the Expand Long Template PCR System (Roche) with non-transformed IR64 as a negative control. PCR was performed in a 20-μL volume containing 1xExpand Long Template buffer 1, 350 μM of each dNTP, 300 nM of each primer, 200 ng of

DNA template, and 1.5 U Expand Long Template Enzyme mix. All primers are listed in Table S8. PCR was performed as follows: 2 min at 94°C, 10 cycles of denaturation at 94°C for 10 s, annealing at 55°C for 30 s, and elongation at 68°C for 4 min; 25 cycles of denaturation at 94°C for 15 s, annealing at 55°C for 30 s, and elongation at 68°C for 4 min. After the last cycle, the samples were incubated for 7 min at 68°C. PCR was performed using a G-Storm GS1 thermal cycler (Gene Technologies Ltd., Essex, UK). Amplification products were excised from the agarose, purified using GeneClean Kit II (QBiogene), cloned into pGEM-T Easy (Promega), and sequenced.

Relative quantification of transcript. Total RNA was isolated from roots and leaves of wild-type and transgenic rice plants using Plant RNA Purification Reagent (Invitrogen, Carlsbad, California, USA) and purified using RNeasy Mini Kit (Qiagen, Valencia, California, USA). Approximately 1 µg of total RNA was used for *DNase*I treatment and cDNA synthesis (using Transcriptor First Strand cDNA Synthesis Kit) as described by the supplier (Roche, Applied Science, Penzberg, Upper Bavaria, Germany). Real-time quantitative RT-PCR (qRT-PCR; LightCycler 480, Roche) was performed using the fluorescent dye SYBR Green (SYBR Select Master Mix, Applied Biosystems) for 40 cycles following the manufacturer's protocol. The transcript level of the OsNAS2 transgene and endogenous OsNAAT1 and OsDMAS1 in transgenic and WT were normalized to the expression level of actin, using the relative standard curve method as previously described[15]. Oligonucleotides ACT1F (5′-CTTCATAGGAATGGAAGCTGCGGGTA-3′) and ACT1R (5′-CGACCACCTTGATCTTCATGCTGCTA-3′)[62] were used to amplify the actin gene, OsNAS2-tg-495F (5′-TCGAGCTCGGTACCTCAG-3′) and OsNAS2-tg-495R (5′-TGCAAGTGCTGCAAGATG-3′) to amplify *OsNAS2*, OsNAAT1-103F (5′-CGGACAAGAACACTACTGCGA-3′) and OsNAAT1-103R (5′-

TTCCTTGCTACCTCTGCCAC-3′) to amplify *OsNAAT1*, and OsDMAS1-126F (5′-CCCCTTCATCACGTCCAAGC-3′) and OsDMAS1-126R (5′-ACAGGCCAGTGCACCAGGTA-3′) to amplify *OsDMAS1*.

NA/DMA detection in polished rice grains using liquid chromatography/electrospray ionization time-of-light mass spectrometry (LC/ESI-TOF-MS). The LC/ESI-TOF-MS method is based on published methods[57,63] with some modifications. One hundred and fifty polished rice grains were obtained from each line and ground to fine powder. Twenty mg of ground rice was weighed into separate Eppendorf tubes and 1,200 mL of sterile distilled water were added to the respective tubes, to release any metal complexed with NA and/or DMA. N ε -nicotyl-lysine (NL) was used as internal standard. The derivatization of NA and DMA involved mixing of 5 µL supernatant (concentrated by evaporation) with 5 µL of sodium borate buffer (1 M, pH 8.0) and 5 µL of EDTA (50 mM, pH 8.0), followed by the addition of 40 µL of FMOC-Cl solution (50 mM) in acetonitrile (CH3CN). The reaction mixture was then heated at 60°C for 30 min. Later, the pH of the reaction mixture solution was adjusted to 4.0 using 5 µL of 5% formic acid to stop the reaction. Quantification was based on the external calibration curve method using the authentic samples of DMA (2.5–80 µM), NA (2.5–80 µM) and NL (20 µM). LC-ESI-TOF-MS measurement was carried out using a JSM-T100LC AccuTOF (JEOL, Tokyo, Japan) in ESI+ mode. The desolvent temperature was 250°C, the orifice 1 temperature was 80°C and the ESI needle voltage was 2,200 V. The LC separation was performed using a Synergi Hydro RP column (4µm, 80 A, 150 × 2.00 mm; Phenomenex, Torrance, CA, USA) with a solvent of 0.5 % formic acid, 36 % water and 63.5 % CH3CN. Using this system, a single run could be completed within 30 min. The detection mass range (m/z) was set from 200 to 1,000.

References

58. Triglia, T., Peterson, M., Kemp, D. A procedure for in vitro amplification of DNA segments that lie outside the boundaries of known sequences. *Nucl Acids Res.* **16**, 81−86 (1988).

59. Ochman, H., Gerber, a S. & Hartl, D. L. Genetic applications of an inverse polymerase chain reaction. *Genetics* **120**, 621–3 (1988).

60. Liu, Y. G., Mitsukawa, N., Oosumi, T. & Whittier, R. F. Efficient isolation and mapping of Arabidopsis thaliana T-DNA insert junctions by thermal asymmetric interlaced PCR. *Plant J.* **8**, 457–63 (1995).

61. Liu, Y. & Huang, N. Efficient amplification of insert end sequences from bacterial artificial chromosome clones by thermal asymmetric interlaced PCR. *Plant Mol. Biol. Rep*. **16**, 175–181 (1998).

62. Li, Q., Sun, S., Yuan, D., Yu, H., Gu, M. & Liu, Q. Validation of candidate reference genes for the accurate normalization of real-time quantitative RT-PCR data in rice during seed development. *Plant Mol. Biol. Rep*. **28**, 49-57 (2010).

63. Wada, Y., Yamaguchi, I., Takahashi, M., Nakanishi, H., Mori, S., & Nishizawa, N. K. Highly sensitive quantitative analysis of nicotianamine using LC/ESI-TOF-MS with an internal standard. *Bioscience, Biotechnology, and Biochemistry* **71**, 435–441 (2007).
